# Dextromethorphan-Bupropion-Esketamine Triadic Pharmacology: Molecular Targets, Enzymatic Interactions, and Risk-Benefit Assessment of a Three-Agent Combination for Treatment-Resistant Depression

**DOI:** 10.3390/ijms272110036

**Published:** 2026-11-09

**Authors:** Andrea Fagiolini, Alessandro Cuomo, Giulio Corrivetti, Mario Pinzi, Pietro Carmellini, Alessandra Marenna, Francesco Monaco, Annarita Vignapiano, Eva Frigola-Capell, the OPADE Group

**Affiliations:** 1 Department of Molecular and Developmental Medicine, University of Siena, Viale Bracci 16, 53100 Siena, Italy; alessandro.cuomo@unisi.it (A.C.); mario.pinzi@student.unisi.it (M.P.); pietro.carmellini@unisi.it (P.C.); 2 European Biomedical Research Institute of Salerno, EBRIS, Via Salvatore De Renzi 50, 84125 Salerno, Italy; g.corrivetti@ebris.eu (G.C.); a.marenna@ebris.eu (A.M.); fmonaco1980@gmail.com (F.M.); annarita.vignapiano@gmail.com (A.V.); 3 Mental Health and Addictions Research Group, Institut d’Investigació Biomèdica de Girona (IDIBGI-CERCA), Departament de Salut, Generalitat de Catalunya, Parc Hospitalari Martí i Julià, Edifici M2, 17190 Salt, Spain; eva.frigola.ias@gencat.cat

**Keywords:** dextromethorphan, dextrorphan, bupropion, esketamine, NMDA receptor, sigma-1 receptor, CYP2D6, CYP2B6, pharmacokinetic boosting, pharmacogenomics

## Abstract

Dextromethorphan is extensively O-demethylated by cytochrome P450 2D6 (CYP2D6) to dextrorphan, and the two molecules do not share a target profile. Dextrorphan is the more potent N-methyl-D-aspartate (NMDA) channel blocker; the parent compound is the more potent serotonin transporter inhibitor; their sigma-1 affinities are comparable, so differences in sigma-1 engagement depend on exposure rather than intrinsic affinity. CYP2D6 activity therefore does not scale exposure to a fixed pharmacology; it determines which pharmacology is expressed. This review develops that principle and extends it to a three-agent system. From published unbound concentrations and functional potencies, we calculate a model-derived fractional-target-engagement index. Under the base-case inputs, CYP2D6 inhibition raises the serotonergic index approximately fourfold while reducing the NMDA channel index; deterministic sensitivity analysis shows that the direction is robust to one-at-a-time 0.5-fold to 2-fold perturbations but not to all joint extreme assumptions. Quinidine and bupropion are analyzed as mechanistically distinct proofs of concept, and quinidine-derived exposure estimates are not treated as quantitative surrogates for the dextromethorphan–bupropion steady state. The framework is then applied to the untested question of whether a boosted dextromethorphan combination and esketamine are redundant or complementary. The agents share an NMDA channel mechanism, while CYP2B6 pathway overlap remains a hypothesis-generating observation rather than evidence of clinically relevant competition. No controlled data on the three-agent combination exist, and no therapeutic recommendation is made.

## 1. Introduction

Dextromethorphan (3-methoxy-17-methylmorphinan) is the dextrorotatory enantiomer of the codeine analogue levomethorphan. The stereochemical inversion abolishes appreciable agonism at the mu-opioid receptor while preserving the morphinan scaffold, and this single structural fact has allowed the molecule to occupy an unusual pharmacological niche: it is an opioid-derived compound without appreciable opioid receptor agonism, whose principal actions lie instead at ionotropic glutamate channels, an intracellular chaperone protein, and monoamine transporters.

The molecule entered clinical use in 1958 as an antitussive, an indication now understood to depend at least in part on antagonism at alpha3beta4 nicotinic acetylcholine receptors and on sigma-1 receptor engagement rather than on a purely central cough reflex mechanism [1,2]. Interest in its central actions expanded substantially after the demonstration, in the late 1980s and early 1990s, that dextromethorphan and its principal metabolite bind within the NMDA receptor channel at a site overlapping the noncompetitive antagonist domain and confer neuroprotection in models of excitotoxic injury [3,4]. Subsequent characterization identified high-affinity binding at the sigma-1 receptor, inhibition of the serotonin and norepinephrine transporters, and activity at additional targets, including voltage-gated sodium and calcium channels and microglial NADPH oxidase [2,5,6].

This accumulation of targets is often described as polypharmacology, a term that implies a single molecule acting simultaneously at several sites [2]. In the case of dextromethorphan, the description is incomplete because the administered molecule is not necessarily the principal unconjugated species present. Dextromethorphan undergoes extensive and rapid O-demethylation by CYP2D6 to dextrorphan, with substantial first-pass extraction; in extensive metabolizers, plasma concentrations of the parent compound after conventional dosing are low and short-lived, while dextrorphan and its glucuronide dominate systemic exposure [7,8,9]. The two species differ most clearly at the NMDA receptor channel and serotonin transporter: dextrorphan is substantially more potent at the NMDA channel, whereas the parent is more potent at serotonin reuptake inhibition. Their sigma-1 binding affinities are comparable in the cited assay, and their functional norepinephrine uptake potencies are similar [2,5,10,11,12].

The practical consequence is that CYP2D6 activity is not a pharmacokinetic detail layered on top of a fixed pharmacodynamic profile. It is a determinant of the pharmacodynamic profile itself. A given oral dose produces materially different patterns of target engagement in a poor metabolizer, an extensive metabolizer, and an extensive metabolizer co-treated with a CYP2D6 inhibitor. This is the molecular rationale that underlies every clinically deployed dextromethorphan combination, and it is the organizing principle of this review.

Section 2 describes the literature search and source selection methods. We then characterize the target profile of the parent compound (Section 3) and its metabolites (Section 4), examine the metabolic shift between them (Section 5), and review downstream signaling (Section 6). Section 7 and Section 8 address disposition and CYP2D6-directed boosting, Section 9 considers CYP2D6 allelic variation, and Section 10 evaluates the mechanistic overlap with esketamine. Throughout, the focus is on molecular mechanisms; clinical efficacy data are cited only where they constrain mechanistic interpretation.

## 2. Literature Search Strategy and Source Selection

### 2.1. Search Strategy and Information Sources

The literature search was initially conducted in PubMed on 12 July 2026 and updated on 30 September 2026. For this revision, the comprehensive query and all sixteen targeted subqueries were rewritten as fully specified strings and independently rerun on 4 November 2026 through the NCBI ESearch API. Publication dates were limited to 1 January 2010 through 30 September 2026, and English-language and human-indexed records were retained. Complete strings, query translations, query-level yields, and PMID membership are provided in the Supplementary Materials.

**Figure 1:**
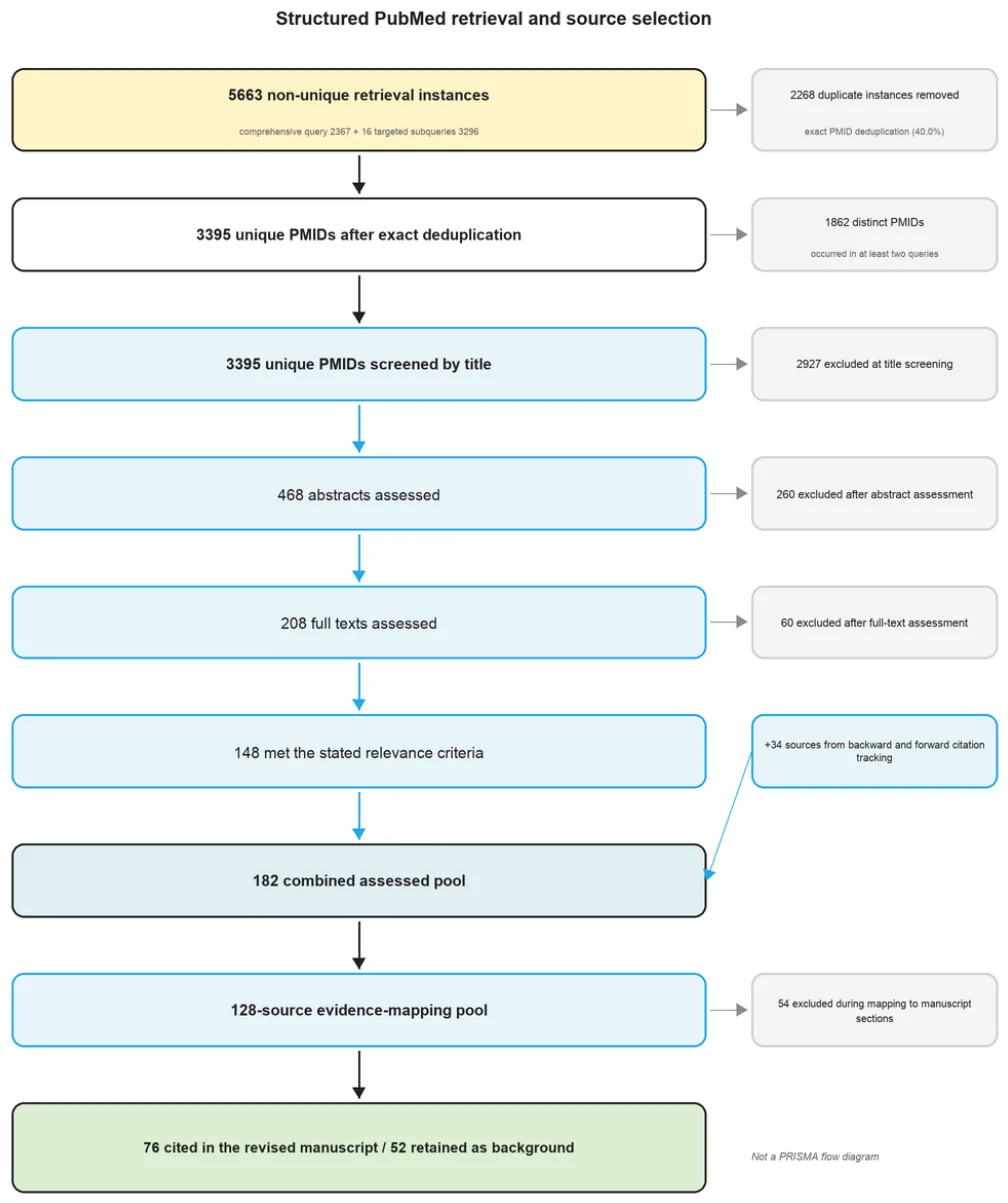
The source identification and selection for the structured narrative review. The comprehensive query returned 2367 records and the sixteen targeted subqueries returned 3296, giving 5663 non-unique retrieval instances in the 4 November 2026 audit. Searches were restricted to publications dated through 30 September 2026 in the English language and human-indexed records. Exact PMID deduplication removed 2268 surplus retrieval instances (40.0%), leaving 3395 unique PMIDs. The count of 1862 refers to distinct PMIDs retrieved by at least two queries, whereas 2268 counts all retrieval instances removed beyond the first occurrence of each PMID. All 3395 unique PMIDs were screened by title; 2927 were excluded and 468 abstracts were assessed. Abstract assessment excluded 260 records, leaving 208 full texts for assessment. Sixty full texts were excluded and 148 met the stated relevance criteria. Backward and forward citation tracking added 34 earlier or preclinical foundational sources, producing a combined assessed pool of 182. Evidence mapping to manuscript sections excluded 54 sources, leaving a 128-source evidence-mapping pool; 76 sources are cited in the revised manuscript and 52 are retained as background. This is not a PRISMA flow diagram.

The search was designed to improve the transparency and breadth of source identification for a mechanistically oriented narrative review. It was not intended to be an exhaustive systematic search, did not include formal risk-of-bias or certainty-of-evidence assessment, and was not used to generate pooled effect estimates. Accordingly, the source selection diagram (Figure 1) is not presented as a PRISMA flow diagram.

The date, language, and human-indexing limits applied to the structured PubMed queries, not to the full mechanistic evidence base. Earlier and preclinical foundational studies were identified by backward and forward citation tracking from key mechanistic reviews and primary studies and then retained when they directly informed molecular targets, channel kinetics, enzyme mechanisms, or assay interpretation. This supplementary route was necessary because a human-indexed 2010–2026 search cannot capture the older experimental literature on which several mechanistic conclusions depend. All 3395 unique PMIDs were screened by title; 2927 were excluded and 468 abstracts were assessed. Abstract assessment excluded 260 records, leaving 208 full texts for assessment; 60 full texts were excluded and 148 met the stated relevance criteria. Citation tracking added 34 sources, producing a combined assessed pool of 182.

#### Supplementary Targeted Search for Section 11, Section 12, Section 13, Section 14 and Section 15 (Enzymatic Interactions, Risk–Benefit Assessment, Special Populations, and Research Agenda)

To address the enzymatic interactions, risk–benefit assessment, contraindications, and mechanistic research agenda presented in Section 11, Section 12, Section 13, Section 14 and Section 15, eight targeted subqueries were run alongside the eight supporting Section 3, Section 4, Section 5, Section 6, Section 7, Section 8, Section 9 and Section 10: CYP2D6-CYP2B6 interactions (*n* = 56); esketamine safety profile (*n* = 746); bupropion toxicity and cardiovascular safety (*n* = 893); dextromethorphan abuse liability (*n* = 130); NMDA-antagonist drug interactions and serotonin toxicity (*n* = 46); antidepressant therapeutic drug monitoring and pharmacogenomics (*n* = 170); special populations and contraindications (*n* = 291); and NMDA plasticity and sigma-1 receptor mechanisms (*n* = 184). Every subquery was anchored to dextromethorphan, dextrorphan, esketamine, ketamine, or bupropion. The sixteen subqueries returned 3296 non-unique hits and were combined with the explicitly field-tagged comprehensive query (*n* = 2367), giving 5663 retrieval instances. Exact PMID deduplication removed 2268 duplicate instances (40.0%), leaving 3395 unique PMIDs; 1862 PMIDs occurred in more than one query. Sources were selected narratively against explicit relevance criteria: CYP450 enzymatic interactions and pharmacokinetic substrate overlap; safety, adverse effects, and contraindications in treatment-resistant depression; drug interactions and therapeutic drug monitoring; mechanistic and translational evidence supporting testable predictions; and special populations, including pregnancy and renal, hepatic, or seizure comorbidity.

### 2.2. Relevance Criteria and Source Selection

The review focused on molecular pharmacology: receptor and transporter binding and functional data for dextromethorphan, dextrorphan, and the downstream morphinan metabolites; downstream signaling consequences of sigma-1 and NMDA receptor engagement; the enzymology of CYP2D6-mediated disposition; the molecular basis of enzymatic boosting; and CYP2D6 pharmacogenomics. In vitro and preclinical evidence was prioritized. Clinical trial evidence was retained only where it constrains mechanistic interpretation, principally studies establishing the magnitude of the exposure change produced by CYP2D6 inhibition. Sources reporting clinical efficacy without mechanistic or exposure data were not retained for detailed assessment.

The comprehensive query and sixteen targeted subqueries returned 5663 non-unique retrieval instances (2367 and 3296, respectively) in the 4 November 2026 audit. Exact PMID cross-checking removed 2268 duplicate instances (40.0%), leaving 3395 unique PMIDs. The audit preserves every full query string, PubMed query translation, query-level PMID list, and deduplicated PMID membership table. All 3395 unique PMIDs were screened by title; 2927 were excluded and 468 abstracts were assessed. Of these, 260 were excluded after abstract assessment and 208 full texts were assessed; 60 full texts were excluded and 148 met the stated relevance criteria. Backward and forward citation tracking added 34 sources, giving a combined assessed pool of 182. Evidence mapping to manuscript sections excluded 54 sources and retained 128; 76 are cited in the revised manuscript and 52 are retained as background. Figure 1 presents the reconciled aggregate downstream flow. The PubMed Search Audit Workbook provides the reproducible search record.The tapentadol pharmacology source added during peer review is included among the 76 cited sources.

### 2.3. Data Abstraction and Citation Checking

Data were abstracted on compound and metabolite identity; target and assay system, including radioligand, functional readout, species, and tissue or cell line; reported affinity or potency values with the conditions under which they were obtained; enzyme kinetic parameters and inhibition constants; allele frequencies and phenotype assignments; and, for exposure studies, the magnitude and direction of change in parent and metabolite concentrations.

## 3. Molecular Pharmacology of Parent Compound

### 3.1. NMDA Receptor Channel Block

Dextromethorphan acts as an uncompetitive, open-channel blocker at the NMDA receptor, binding within the pore at a site overlapping the domain labeled by phencyclidine and MK-801 [2,3]. Binding requires prior channel activation by glutamate and a co-agonist, which confers use dependence: block accumulates preferentially at receptors undergoing sustained activation [3]. Affinity is modest. Radioligand displacement at the MK-801 site gives Ki values of 962 nM and 2120 nM, depending on the radioligand concentration, and voltage-clamp recording in cultured neocortical neurons gives an IC50 of 550 nM [2]. Crucially, dextromethorphan is a low-trapping blocker: in a directly comparable patch-clamp series, its dissociation rate constant is 0.179 per second, faster than those of dextrorphan (0.075), ketamine (0.075) and MK-801 (0.005) and closest to that of memantine (0.200) [2,13]. This kinetic profile, rather than potency, is what distinguishes it from the dissociative channel blockers and predicts preferential action at tonically active rather than phasically recruited receptors.

Subunit selectivity has been incompletely characterized. Available data do not support strong GluN2B selectivity of the type described for ifenprodil-site ligands, and the pore-directed binding mode predicts limited discrimination among GluN2 subunits. However, mutagenesis work indicates that the molecular determinants of dextrorphan binding are not identical to those of MK-801: residues N616 and A627 of GluN1 are critical for MK-801 but not for dextrorphan, whereas W611 and N812 are required for dextrorphan, placing its recognition site shallower within the channel vestibule [10]. This remains an open question worth explicit statement rather than assumption.

### 3.2. Sigma-1 Receptor

Dextromethorphan binds the sigma-1 receptor with a Ki of 150 nM against 3H-pentazocine, its second-highest affinity after the serotonin transporter, and shows only weak affinity at sigma-2 sites (Ki approximately 11 microM) [2,11]. It is important to state at the outset, because it constrains the argument developed in Section 5, that dextrorphan binds sigma-1 with comparable affinity (Ki 118 nM) [2,11]. Sigma-1 is therefore not a site at which parent and metabolite differ appreciably, and any difference in sigma-1 engagement between metabolic conditions must arise from exposure rather than from affinity.

The sigma-1 receptor is not a conventional receptor but a ligand-operated chaperone resident at the mitochondria-associated endoplasmic reticulum membrane (MAM), where it regulates inositol trisphosphate receptor type 3 stability and calcium flux into mitochondria, and from which it translocates upon activation to modulate ion channels and signaling complexes at the plasma membrane [14,15]. Classification of dextromethorphan as an agonist rests on the attenuation of its antitussive, anticonvulsant, neuroprotective and antidepressant-like effects by selective sigma-1 antagonists such as BD1047 and BD1063, rather than on a direct functional assay [2]. A caveat carried through this review is that the analysis in [2] leaves open whether unbound brain concentrations achieved clinically are sufficient for functional sigma-1 agonism, as opposed to mere occupancy: agonist-dependent translocation from the MAM has been described as requiring concentrations around ten times the binding Ki.

Because sigma-1 affinity is considerably higher than NMDA affinity for the parent compound, the concentration range over which the parent is present determines whether sigma-1 engagement occurs in relative isolation or alongside channel block. This is directly relevant to the metabolic argument developed in Section 5.

### 3.3. Monoamine Transporters

The serotonin transporter is the highest-affinity target identified for the parent compound, with a binding Ki of 40 nM and a functional Ki of 23 nM for inhibition of serotonin uptake into rat brain synaptosomes [2,11,12]. Dextrorphan is roughly seventeen-fold weaker functionally (Ki 401 nM) [2,12]. At the norepinephrine transporter, the picture differs: radioligand binding suggests very weak interaction for both species (Ki approximately 6000 nM), whereas functional uptake inhibition gives Ki values of 240 nM for dextromethorphan and 340 nM for dextrorphan, a discrepancy of more than an order of magnitude whose basis is not established [2,12]. The functional values are the ones relevant to the exposure comparison, and on those values, norepinephrine reuptake inhibition is only modestly parent-weighted.

The serotonin transporter is thus the clearest point of divergence between the two species after the NMDA channel, and it diverges in the opposite direction. This pairing, not the sigma-1 receptor, is what carries the argument of Section 5.

### 3.4. Nicotinic Acetylcholine Receptors

Dextromethorphan is a functional antagonist at alpha3beta4 nicotinic acetylcholine receptors with an IC50 of 700 nM in Xenopus oocytes, with approximately 5-fold selectivity over alpha4beta2 receptors (IC50 3900 nM); dextrorphan is roughly 2-fold weaker at alpha3beta4 (IC50 1300 nM) [2,16]. Antagonism is noncompetitive with respect to the agonist site, since neither compound displaces epibatidine binding [2]. This subtype is expressed presynaptically on noradrenergic terminals and mediates acetylcholine-evoked catecholamine release from adrenal chromaffin cells, and its blockade has been linked to the antitussive action and, in preclinical models, to reduced drug-seeking behavior [2]. The potency and selectivity profile resembles that of mecamylamine.

### 3.5. Opioid Receptors and Other Targets

Affinity at mu-, delta- and kappa-opioid receptors is absent at screening concentrations: dextromethorphan produced no significant displacement at 10,000 nM in a panel covering all three [2]. This is consistent with the stereochemical basis of the compound and justifies excluding opioid mechanisms from further consideration. Weak interactions were detected at adrenergic alpha1D (Ki 830 nM) and alpha1A (Ki 3000 nM) receptors, and dextromethorphan blocks a range of voltage-gated sodium, potassium and calcium channels and 5-HT3 and glycine-gated channels with IC50 values from 3000 nM upward, all well above achievable unbound brain concentrations, and is therefore unlikely to contribute at therapeutic doses [2]. Inhibition of microglial NADPH oxidase 2 is a separate case, since it has been demonstrated in vivo at ultra-low doses in models of neuronal injury [6]. The principal binding, functional-potency, and channel kinetic values are summarized in Table 1.

Note on interpretation: Exposure values are tabulated separately in Table 2 because the steady-state and single-dose regimens are not interchangeable. Taking the 30 mg single-dose comparison as the reference regimen, unbound dextromethorphan under CYP2D6 inhibition reaches 66 nM, which exceeds its serotonin reuptake Ki of 23 nM by nearly threefold, approaches its norepinephrine reuptake Ki of 240 nM, and reaches a fraction of its sigma-1 binding Ki of 150 nM and its NMDA channel IC50 of 550 nM. Without an inhibitor, unbound dextromethorphan is 3.7 nM, and none of these thresholds is approached, so the pharmacology is carried by dextrorphan, whose NMDA channel IC50 of 72 nM is the lowest functional value for either species. The analysis in [2] concludes on this basis that the boosted combination inhibits serotonin and norepinephrine reuptake and produces the fractional block of NMDA and alpha3beta4 channels, while it remains unclear whether concentrations sufficient for functional sigma-1 agonism are reached. Section 5.1 converts these comparisons into model-derived fractional-engagement indices.

## 4. Dextrorphan and the Downstream Metabolites

### 4.1. Metabolic Scheme

Dextromethorphan undergoes two principal phase I transformations: O-demethylation at position 3, catalyzed predominantly by CYP2D6, yields dextrorphan (3-hydroxy-17-methylmorphinan); N-demethylation at position 17, catalyzed mainly by CYP3A4 with substantial contributions from CYP2B6 and lesser contributions from other isoforms, yields 3-methoxymorphinan [7,8,19]. Both products can undergo the complementary reaction to give the doubly demethylated 3-hydroxymorphinan [7]. Dextrorphan is rapidly conjugated by UDP-glucuronosyltransferases to dextrorphan-O-glucuronide, which circulates at concentrations far exceeding those of any unconjugated species [2].

The kinetic asymmetry between these routes is the central point. CYP2D6-mediated O-demethylation is a high-affinity reaction with a Km in the low micromolar range, reported at approximately 7 micromolar in adult human liver microsomes, and it is the dominant clearance pathway at therapeutic concentrations in extensive metabolizers [7]. N-demethylation is a low-affinity, higher-capacity route with a Km two orders of magnitude greater, reported in the range of 230 to 650 micromolar, which becomes proportionally more important when CYP2D6 is absent, saturated, or pharmacologically inhibited [7,8]. Dextromethorphan disposition is therefore not simply slowed by CYP2D6 inhibition, it is rerouted, with consequences for the metabolite mixture present at the target sites.

### 4.2. Dextrorphan

Dextrorphan is the more potent species at the NMDA receptor channel by every measure: the binding Ki is 148 nM, against 962 nM for the parent in the same assay, the functional IC50 is 72 nM, against 550 nM in cultured neocortical neurons, and the patch-clamp IC50 is 1300 nM, against 6100 nM [2,13,17]. Radioligand studies establish that its binding localizes to the noncompetitive antagonist domain of the activated receptor–channel complex, is profoundly glutamate- and glycine-dependent, and is distributed across forebrain regions in a pattern matching NMDA receptor distribution rather than that of sigma sites [3,4]. Its dissociation kinetics are also slower than those of the parent (Koff 0.075 against 0.179 per second), placing it closer to ketamine on the trapping continuum, and dextrorphan, unlike dextromethorphan, produces phencyclidine-like increases in locomotor activity in rodents [2,13]. In extensive metabolizers, it is the species principally responsible for glutamatergic effects after oral dosing.

The divergence runs the other way at the serotonin transporter, where dextrorphan is approximately seventeen-fold weaker than the parent (functional Ki 401 nM versus 23 nM) [2,12]. At the norepinephrine transporter, the two are close (340 nM versus 240 nM), and at the sigma-1 receptor, dextrorphan is, in the cited assay, marginally the higher-affinity ligand (Ki 118 nM versus 150 nM) [2,11,12]. The metabolite is therefore shifted specifically toward NMDA channel block and away from serotonin reuptake inhibition, while sigma-1 binding and functional noradrenergic potency are broadly comparable.

A further consideration, insufficiently addressed in the literature, concerns central nervous system penetration. Dextrorphan is more polar than the parent compound and is extensively glucuronidated; the relationship between plasma dextrorphan concentrations and unbound brain concentrations of the active species is therefore not straightforward, and inferences about central target engagement drawn from plasma metabolite ratios should be qualified accordingly.

### 4.3. 3-Methoxymorphinan and 3-Hydroxymorphinan

These metabolites are generally treated as pharmacologically minor and have been characterized far less thoroughly. 3-Hydroxymorphinan retains measurable activity at NMDA and sigma sites and has been reported to possess neurotrophic and anti-inflammatory properties in preclinical models, raising the possibility that it contributes to effects observed in vivo that are not accounted for by the parent and dextrorphan alone [2]. 3-Methoxymorphinan, whose formation increases when CYP2D6 is inhibited [7], has received minimal pharmacological characterization despite becoming a proportionally larger fraction of the metabolite pool precisely under the conditions created by boosting strategies. This is a concrete and citable gap in the field.

## 5. Metabolism as Qualitative Switch in Target Profile

**Figure 2:**
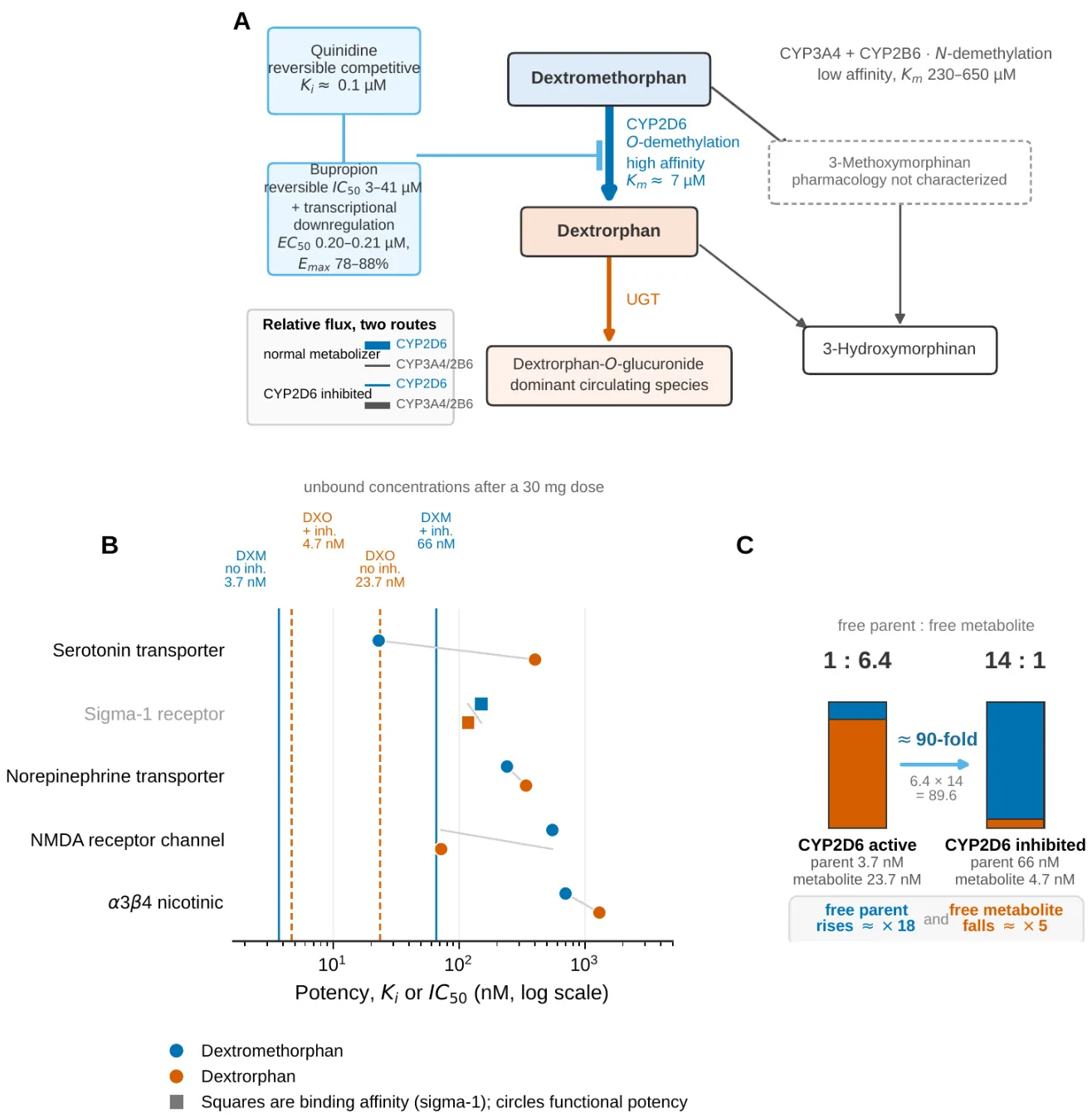
The metabolic routing and functional target profiles of dextromethorphan and dextrorphan. Panel (**A**) shows the CYP2D6-dependent O-demethylation and secondary N-demethylation pathways. Panel (**B**) compares the functional potencies and sigma-1 binding affinities with the unbound-dextromethorphan range during CYP2D6 inhibition. Panel (**C**) illustrates the change in the free parent-to-metabolite ratio. The sigma-1 affinities are comparable and do not drive the inversion. Values and sources are listed in Table 1; unbound plasma concentrations are assumed to approximate brain extracellular concentrations.

CYP2D6 inhibition not only increases and stabilizes exposure to the parent compound but also changes the composition of the active moiety. Figure 2 illustrates the metabolic routing, target profiles, and parent-to-metabolite shift produced by CYP2D6 inhibition.

For the parent compound: serotonin reuptake (23 nM) > norepinephrine reuptake (240 nM) > NMDA channel (550 nM) > alpha3beta4 (700 nM). For dextrorphan: NMDA channel (72 nM) > norepinephrine reuptake (340 nM) > serotonin reuptake (401 nM) > alpha3beta4 (1300 nM) [2,12,16,17]. The extremes of the two orderings are exchanged: the parent’s most potent action is its metabolite’s third, and the metabolite’s most potent action is the parent’s third. The sigma-1 binding affinities are comparable for both (150 and 118 nM) and therefore do not participate in the inversion, although the amount of sigma-1 engagement still depends on how much total unbound drug is present.

It follows that the ratio of parent to metabolite is not a pharmacokinetic descriptor but a pharmacodynamic one, and the magnitude of the shift is large. With dextromethorphan at 30 mg alone, unbound plasma dextromethorphan reaches 3.7 nM and free unconjugated dextrorphan is approximately 6.4-fold higher, that is, approximately 23.7 nM; with a CYP2D6 inhibitor at the same dose, unbound dextromethorphan reaches 66 nM and exceeds free dextrorphan by approximately 14-fold, placing free dextrorphan at approximately 4.7 nM [2,18]. The ratio of free parent to free metabolite therefore changes by a factor of approximately 90 (6.4 × 14 = 89.6). Two components of that shift deserve a separate statement because only the first is routinely acknowledged: the unbound parent rises approximately 18-fold, and the free metabolite falls approximately 5-fold. The second has not, to the best of our knowledge, been made explicit, and it has direct consequences for glutamatergic target engagement. Set against the potencies above, this moves the exposure from a regime in which no parent-mediated target threshold is approached and dextrorphan carries NMDA channel block into a regime in which serotonin reuptake inhibition is substantial, norepinephrine reuptake inhibition is near-threshold, and NMDA and nicotinic block are fractional and carried increasingly by the parent with its faster off-kinetics [2]. The resulting pharmacology is not an amplified version of the uninhibited condition; it is a different composite.

### 5.1. Fractional Target Engagement

**Figure 3:**
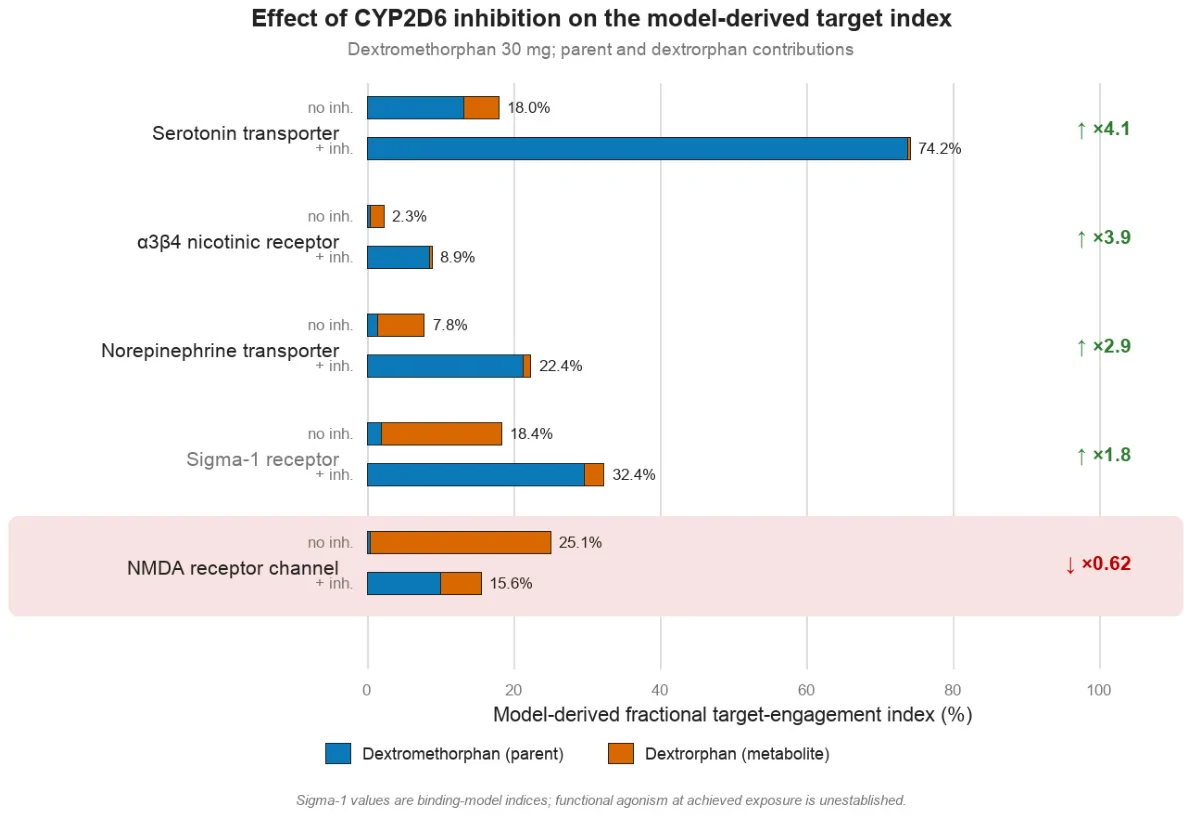
Model-derived fractional target-engagement indices for dextromethorphan and free dextrorphan after a 30 mg dose, with and without CYP2D6 inhibition. Values were calculated from the functional potencies in Table 1 and unbound concentrations in Table 2 using the one-site model stated in the text. The indices are not measured receptor occupancies. The sigma-1 row is a binding-model estimate; functional agonism at these exposures is unestablished. Green arrows indicate an increased index and the red arrow a decreased index after CYP2D6 inhibition.

Table 3 and Figure 3 convert the exposure and potency values into a model-derived fractional-target-engagement index using f = SUM(Ci/Ki)/(1 + SUM(Ci/Ki)), with the unbound plasma concentration used as a proxy for the brain interstitial concentration, as in [2]. This competitive one-site equation is used only as a normalized exposure-to-potency heuristic. It is most interpretable for a within-target comparison when the assay, potency measure, and concentration proxy are held constant. It does not make heterogeneous Ki and IC50 values interchangeable across targets. For the NMDA receptor, the equation does not represent measured receptor occupancy because open-channel probability, agonist and voltage dependence, use-dependent block, trapping, subunit composition, and time-varying exposure are not included. NMDA results are therefore interpreted as conditional direction-of-change estimates under the stated inputs, not as absolute occupancy or predicted clinical effects.

Under the base-case inputs, the NMDA row decreases from 25.1% to 15.6% in the one-site index (a change of −9.5 percentage points). This result should not be stated as a measured reduction in receptor occupancy. It indicates that, within the simplified calculation and the source concentration ratio, the loss of free dextrorphan at the more potent functional value is not fully offset by the increase in the parent compound. The conclusion is therefore that CYP2D6 inhibition changes the modeled composition of NMDA channel engagement rather than uniformly amplifying it.

The principal limitations are assay heterogeneity, the unvalidated unbound-plasma-to-brain proxy, omission of NMDA channel-state and kinetic behavior, and uncertainty in the parent-to-metabolite ratios. A deterministic sensitivity analysis is provided in the Supplementary Calculation Workbook. Varying each concentration or potency input independently from 0.5-fold to 2-fold preserved a negative NMDA difference in all one-at-a-time scenarios (range, −19.91 to −1.38 percentage points; base case, −9.51). In a joint 3 × 3 grid, the sign reversed in one extreme scenario that favored the parent compound 2-fold and dextrorphan 0.5-fold (+6.31 percentage points). Thus, the base-case direction is robust to individual perturbations but not to all joint assumptions about relative brain exposure and potency.

The exposure contrast in Table 2 and Table 3 comes from dextromethorphan with and without quinidine and is used as a controlled proof of CYP2D6 inhibition, not as a quantitative surrogate for the dextromethorphan–bupropion steady state. Quinidine is a direct reversible inhibitor, whereas bupropion combines weaker reversible inhibition with slower transcriptional downregulation and contributes an independent pharmacology. Product-specific dose, accumulation, timing, and metabolite data are therefore required before the numerical estimates can be extrapolated to dextromethorphan–bupropion treatment. In the absence of matched free parent and dextrorphan concentrations for that regimen, no parallel model-derived calculation is presented.

### 5.2. Implications

Several implications follow, and each is testable:

- **Dose escalation and enzymatic boosting are not interchangeable.** Raising the dose of dextromethorphan alone increases both species while preserving the metabolizer-determined ratio and is constrained by saturation kinetics and by tolerability attributable to the metabolite. Inhibiting CYP2D6 changes the ratio. Preclinical and translational studies that treat these as equivalent manipulations of a single exposure variable are testing different pharmacological conditions.
- **Poor metabolizers receiving dextromethorphan alone approximate the boosted condition.** This constitutes a natural experiment against which the pharmacodynamic predictions above can be evaluated, using the metabolic ratio as the grouping variable rather than the genotype-inferred phenotype.
- **Preclinical species differences are not incidental.** Rodents differ substantially from humans in the enzymes mediating dextromethorphan O-demethylation and in their relative capacity. The parent-to-metabolite ratio achieved in a rodent experiment may correspond to no human condition, and comparability should be established on metabolic rather than dose grounds.
- **The active moiety is a mixture whose composition is a controlled variable.** This is uncomfortable for regulatory and mechanistic frameworks built around a single active species, but it is the honest description of the system.

## 6. Downstream Signaling Consequences

### 6.1. The Sigma-1 Receptor as a Ligand-Operated Chaperone

The sigma-1 receptor is a 223-amino-acid protein with a single transmembrane domain and a cupin-like beta-barrel ligand-binding fold, resolved crystallographically as a trimer with the ligand buried at the center of each protomer’s carboxy-terminal domain [20,21]. The binding cavity is large and hydrophobic and shows marked plasticity, accommodating chemically dissimilar ligands in similar positions, which accounts for the unusually broad ligand recognition of this protein [20]. Under basal conditions, the receptor resides at the mitochondria-associated endoplasmic reticulum membrane in a complex with the chaperone BiP. Ligand binding or endoplasmic reticulum calcium depletion dissociates this complex, freeing the receptor to stabilize inositol trisphosphate receptor type 3 and to sustain prolonged calcium transfer from the endoplasmic reticulum to mitochondria, thereby supporting tricarboxylic acid cycle activity and ATP production [14,15].

Upon sustained activation or cellular stress, the receptor translocates to the plasma membrane and to the nuclear envelope, where it modulates voltage-gated potassium, sodium, and calcium channels, interacts with signaling proteins, including Rac1-GTPase and Bcl2, at the mitochondrial membrane, and influences transcriptional programs [14,22]. Sigma-1 activation additionally attenuates the endoplasmic reticulum stress response and modulates mitochondrial bioenergetics: agonists increase complex I activity in a calcium-dependent, antagonist-sensitive manner and produce a moderate rise in reactive oxygen species under physiological conditions while protecting against pathological oxidative stress [23].

**Figure 4:**
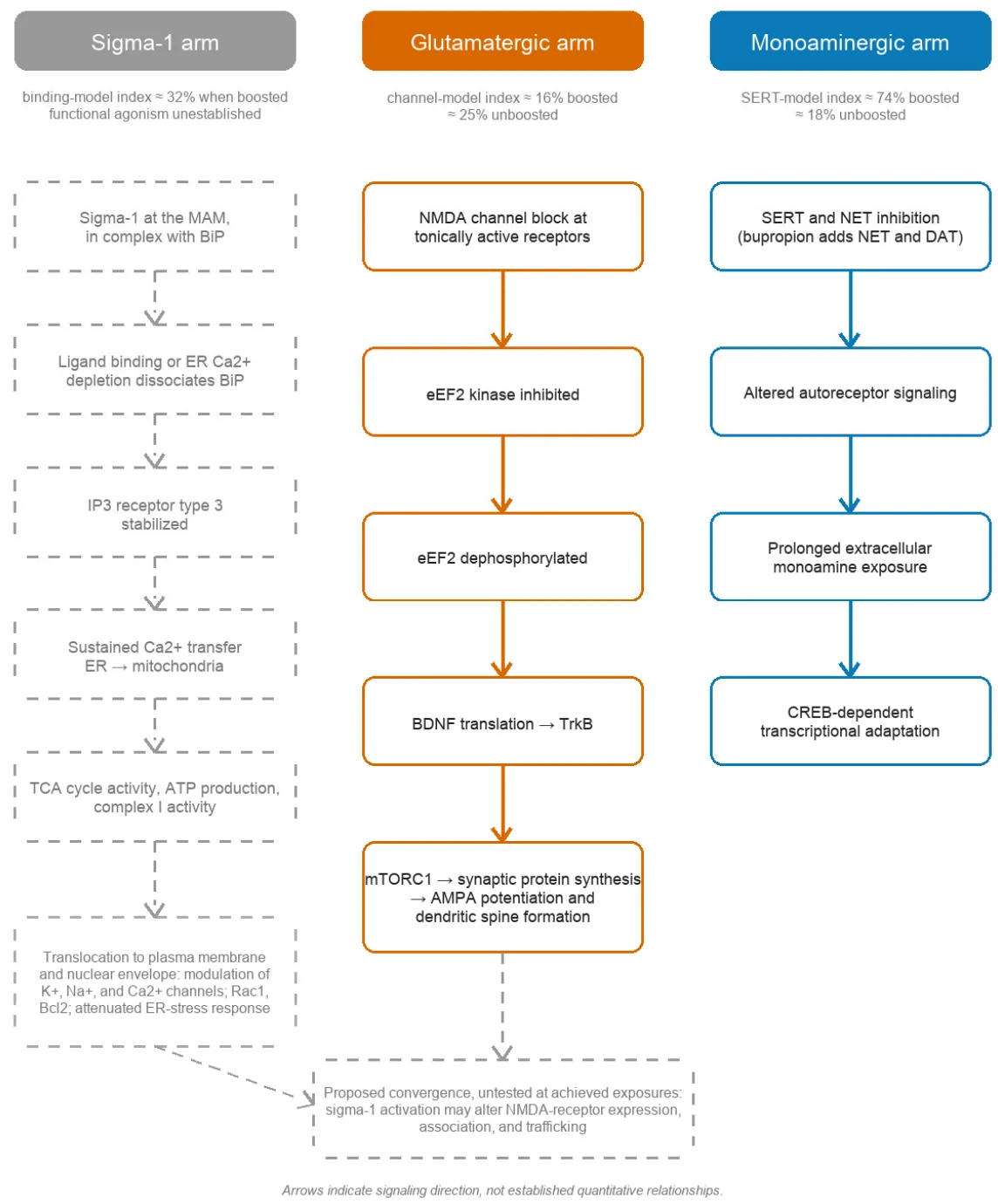
Signaling pathways associated with sigma-1 binding, NMDA open-channel block, and monoamine-transporter inhibition. Solid arrows indicate proposed signaling direction, not an established quantitative relationship. Dashed gray elements are conditional because functional sigma-1 agonism at the modeled exposure is unestablished. Percentage labels in the graphic are model-derived indices, not measured receptor occupancy. MAM, mitochondria-associated endoplasmic reticulum membrane.

These functions place sigma-1 engagement upstream of several processes relevant to neural plasticity: mitochondrial bioenergetics, calcium homeostasis, proteostatic stress, and neuroinflammatory tone. Notably, none of these requires or is explained by NMDA channel block, which supports treating the sigma-1 component as a mechanistically distinct contribution rather than as an adjunct to glutamatergic action. Figure 4 provides a schematic representation of these three signaling arms and their points of convergence.

### 6.2. Glutamatergic Modulation and Plasticity Signaling

Uncompetitive NMDA receptor block at tonically active receptors relieves the suppression that eukaryotic elongation factor 2 kinase exerts on local dendritic protein synthesis, permitting the rapid translation of brain-derived neurotrophic factor (BDNF) [24,25]. Released BDNF engages TrkB, activating cascades that converge on the mechanistic target of rapamycin complex 1 and on synaptic protein synthesis, with the consequent potentiation of AMPA receptor responses and increases in dendritic spine formation [26]. Inhibitors of eukaryotic elongation factor 2 kinase reproduce the rapid behavioral effects of NMDA receptor blockade, identifying this kinase rather than the receptor itself as the proximate molecular substrate [24,26]. Parallel work indicates that ketamine and its active metabolites also regulate STAT3 and type I interferon signaling in human microglia, with downstream effects on BDNF and synaptic proteins, adding an immune arm to this pathway [27]. The parallel pathways engaged by different species within the composite exposure are depicted in Figure 4.

This pathway was characterized principally by ketamine and its metabolites [24,25,26], and the extent to which it is engaged by dextrorphan at achievable exposures has not been established with comparable rigor. In the directly comparable patch-clamp dataset, dextrorphan and ketamine had the same reported dissociation rate constants (0.075 per second), whereas dextromethorphan dissociated faster (0.179 per second) [2,13]. Differences in clinical exposure, potency, route, and depth of channel block therefore should not be attributed to a demonstrated kinetic separation between dextrorphan and ketamine. Direct measurement of eEF2 phosphorylation, BDNF release, mTORC1 activation, and AMPA surface expression under defined parent-to-metabolite ratios would strengthen the mechanistic case.

### 6.3. Convergence and Interaction Between the Two Arms

The sigma-1 and glutamatergic arms are not independent. In vivo administration of selective sigma-1 agonists increases the hippocampal expression of GluN2A and GluN2B subunits and PSD-95, increases the physical interaction between GluN2 subunits and the sigma-1 receptor, and drives the trafficking of NMDA receptors to the cell surface [28]. Sigma-1 activation also supports the mitochondrial function required to sustain plasticity-associated protein synthesis [23]. There is thus a plausible molecular basis for supra-additive interaction between the two components, which is of direct relevance to the argument of Section 5: a composite exposure containing both parent and metabolite may produce signaling outcomes not predicted from either species alone.

This remains a hypothesis. Testing it requires experimental designs in which parent and metabolite concentrations are varied independently rather than jointly, which is achievable in vitro and in preparations where CYP2D6 activity can be manipulated pharmacologically.

### 6.4. Monoaminergic Contribution

Inhibition of SERT and NET engages signaling consequences characteristic of monoamine reuptake inhibition, including altered autoreceptor signaling, prolonged extracellular monoamine exposure, and transcriptional adaptations in CREB-dependent programs. The serotonergic component is strongly parent-weighted, whereas functional norepinephrine uptake potency is similar for parent and metabolite. CYP2D6 inhibition therefore most predictably amplifies the serotonergic component and increases parent-mediated monoaminergic activity rather than switching the entire monoaminergic arm on from zero.

## 7. Enzymatic Basis of Disposition

### 7.1. CYP2D6 Catalysis

CYP2D6 catalyzes O-demethylation of dextromethorphan with high affinity, and the reaction is sufficiently selective and well characterized that dextromethorphan serves as the standard probe substrate for CYP2D6 phenotyping in vivo and the reference reaction in recombinant enzyme panels [7,8]. In human liver microsomes, the reaction is biphasic, but the high-affinity component, inhibited by quinidine with a Ki near 0.1 micromolar, accounts for approximately 96 percent of the total intrinsic clearance by this route [8]. Binding is consistent with the canonical CYP2D6 pharmacophore: a basic nitrogen protonated at physiological pH, forming an ionic interaction with acidic residues in the active site, positioned at a defined distance from the site of oxidation on an aromatic ring. The crystal structure of human CYP2D6 confirms a well-defined active-site cavity above the heme in which Asp301, Glu216, and Phe483 act as substrate-binding residues, with Phe120 controlling the orientation of the aromatic ring found in most substrates [29]. Dextromethorphan satisfies this pharmacophore closely, which accounts for both its high affinity and its utility as a probe.

Reported Km values for the O-demethylation reaction lie in the low micromolar range, approximately 7 micromolar in adult human liver microsomes, well below the concentrations reached in the portal circulation after ordinary oral doses [7]. The reaction is therefore at least partially saturable within the clinically relevant range, which contributes to the greater than dose-proportional increases in parent exposure observed at higher doses.

### 7.2. First-Pass Extraction and the Origin of the Exposure Problem

Oral bioavailability of dextromethorphan in extensive metabolizers is low, owing to extensive presystemic extraction [2,9]. Because CYP2D6 is expressed at high levels in hepatocytes and the O-demethylation reaction is rapid, the fraction of an oral dose reaching the systemic circulation as a parent compound is small and variable: with 60 mg twice daily and no enzyme inhibition, mean plasma concentrations of the parent compound were 12 nanograms per milliliter, with individual values below the limit of quantification in several subjects [9]. Peak parent concentrations are reached rapidly and decline with a short apparent half-life, whereas dextrorphan and its glucuronide persist.

This is the pharmacokinetic problem that boosting strategies exist to solve, and it is worth stating in molecular rather than descriptive terms: a high-affinity, high-capacity hepatic enzyme intercepts the substrate before it reaches its targets and converts it into a species with a different target profile.

### 7.3. Secondary Pathways

CYP3A-mediated N-demethylation to 3-methoxymorphinan is a lower-affinity route that accounts for a minority of the clearance in extensive metabolizers but assumes greater proportional importance when CYP2D6 is inhibited or genetically deficient; in poor metabolizers, N-demethylation is modestly higher than that in extensive metabolizers but does not compensate for the defective O-demethylation pathway [7]. The pathway is not attributable to CYP3A alone: selective CYP3A inhibition reduces 3-methoxymorphinan formation by only about half, and CYP2B6 contributes substantially to the residual activity, with lesser contributions from other isoforms [8,19]. For this reason, the N-demethylation ratio is an imperfect index of CYP3A activity in its simplest form, and composite indices incorporating 3-hydroxymorphinan correlate better with midazolam clearance [30]. Glucuronidation of dextrorphan is rapid and represents the principal elimination route for the metabolite; polymorphism at the relevant UGT loci introduces an additional and generally neglected source of interindividual variability in circulating, unconjugated dextrorphan.

An underappreciated consequence of CYP2D6 inhibition is therefore metabolic rerouting: the proportion of the dose handled by CYP3A4 rises, and with it so too does exposure to 3-methoxymorphinan, a species whose pharmacology is essentially uncharacterized. Any complete molecular account of a boosted combination should address this shift rather than treating the metabolite pool as though it consisted only of dextrorphan.

### 7.4. The Metabolic Ratio as a Molecular Biomarker

The plasma or urinary ratio of dextromethorphan to dextrorphan provides a direct, continuous index of functional CYP2D6 activity, integrating genotype, enzyme induction or inhibition, hepatic function, and substrate competition; its frequency distribution in population samples is bimodal, reflecting the polymorphic contribution of CYP2D6 to the O-demethylation reaction [7]. For the purposes of this review, its value is more specific: because the two species have divergent target profiles, the metabolic ratio is a proxy for the composition of the composite pharmacological exposure, not merely for clearance. Studies that stratify on genotype alone discard this information; studies that report metabolic ratios can relate molecular outcome measures directly to the parameter that determines target engagement [31,32].

## 8. Enzyme Inhibition as a Molecular Design Principle

### 8.1. Two Established Proofs of Concept

Two CYP2D6-directed strategies have been developed as components of fixed combinations with dextromethorphan. They turn out to work by different mechanisms, and the difference is more instructive than the similarity of their clinical effects. The molecular characteristics of these and alternative approaches are compared in Table 4.

Quinidine is a potent, reversible, competitive CYP2D6 inhibitor, substantially more potent as an inhibitor than as a substrate, with a Ki against dextromethorphan O-demethylation in human liver microsomes near 0.1 micromolar and reported at 0.41 micromolar in recombinant systems [8,33]. The original demonstration of the principle is unambiguous: in patients receiving 60 mg dextromethorphan twice daily, addition of 75 mg quinidine twice daily raised mean plasma concentrations of the parent compound from 12 to 241 nanograms per milliliter, an approximately 20-fold increase, accompanied by an increased occurrence of adverse effects in some patients [9]. The combination was subsequently developed and approved for pseudobulbar affect [36,37]. The limitation of quinidine is not its enzymology, which is close to ideal, but its intrinsic cardiac electrophysiological activity, including potassium channel block and QT prolongation, which is unrelated to and undesired alongside the enzymatic function; dose reduction of the quinidine component was pursued specifically to mitigate this liability [36].

Bupropion presents a more complicated and, mechanistically, more interesting case. It is a strong CYP2D6 inhibitor in vivo, increasing desipramine exposure 5.2-fold and producing phenoconversion from an extensive to poor metabolizer phenotype, as judged by the dextromethorphan-to-dextrorphan urinary ratio [31,32]. Yet, in vitro, it is only a weak reversible inhibitor: in human liver microsomes, with dextromethorphan itself as the probe substrate, IC50 values are 3 to 41 microM across bupropion and its circulating metabolites, and inhibition is markedly stereoselective, S-bupropion being 14-fold more potent than R-bupropion [32]. Independent work gives IC50 values of 58 and 74 microM for inhibition of dextromethorphan O-demethylation by bupropion and hydroxybupropion, respectively, confirming low reversible inhibitory potency against CYP2D6 [38]. Accounting for all species at their measured steady-state unbound concentrations predicts only 43 percent inhibition of CYP2D6, a greater than 5-fold underprediction of the observed effect, and hepatic partitioning does not close the gap because IC50 values in suspension hepatocytes match those in microsomes [32].

The resolution is that bupropion and its metabolites downregulate CYP2D6 at the transcriptional level. In HepG2 cells and plated human hepatocytes, they reduce CYP2D6 mRNA concentration-dependently, with EC50 values of 0.21 microM for bupropion and 0.20 microM for S,S-hydroxybupropion and maximal reductions of 78 to 88 percent, with corresponding falls in CYP2D6 protein; the effect is specific, since CYP3A4 and CYP1A2 mRNA are unaffected, and it does not proceed through the small heterodimer partner pathway by which retinoic acid represses CYP2D6 [32]. Combining reversible inhibition with predicted 68 percent downregulation accounts for an 82 percent decrease in CYP2D6 activity, against the approximately 90 percent observed [32]. This is described by its authors as the first clinical drug interaction attributable to the downregulation of a cytochrome, P450, and the first CYP2D6 interaction arising from transcriptional regulation. The 2021 corrigendum to Reference [32] corrected expression-level data; numerical estimates from the original figures should therefore not be interpreted independently of that correction.

The contrast with quinidine is therefore sharper than a comparison of inhibition constants would suggest, and it is a contrast of mechanism class rather than of potency alone. Quinidine achieves boosting through the direct, potent, reversible occupancy of the active site, with an effect that tracks inhibitor concentration and resolves as the inhibitor is eliminated. Bupropion achieves comparable net enzyme suppression largely by reducing how much enzyme is present, an effect whose onset and offset follow mRNA and protein turnover rather than inhibitor kinetics and which therefore requires days to establish and to reverse. In the plated hepatocyte experiments, the apparent inhibition rose from 22 to 63 percent after one hour to 60 to 93 percent after 72 h at unchanged inhibitor concentrations [32]. This temporal signature has practical consequences for dose titration, for the interpretation of early-treatment pharmacokinetics, and, as Section 10.4 argues, for what happens when a third agent is added.

Bupropion also differs from quinidine in not being pharmacodynamically silent: it contributes norepinephrine and dopamine reuptake inhibition and nicotinic antagonism and lowers seizure threshold [34]. The phase 2 trial of the combination addressed the attribution problem directly by using sustained-release bupropion as the active comparator rather than a placebo [39]; the phase 3 trial used a placebo comparator [40]. Attribution of effect between the enzymatic and pharmacodynamic contributions of such an agent remains non-trivial and is further complicated by the fact that the enzymatic contribution is itself of two kinds operating on different timescales.

### 8.2. Molecular Criteria for a Boosting Partner

Framing the requirement at the level of enzyme kinetics rather than therapeutic class yields a definable set of criteria:

- **Potency, if the mechanism is reversible inhibition.** An inhibition constant low enough that effective inhibition occurs at doses below those producing the agent’s own primary pharmacology is required. Quinidine shows that a Ki near 0.1 microM permits this separation; bupropion shows that IC50 values of 3 to 41 microM do not, which is why its in vivo effect cannot be reversible inhibition alone.
- **Mechanism class, which sets the temporal behavior.** Reversible competitive inhibition produces an effect that tracks inhibitor concentration and resolves on elimination. Mechanism-based inactivation produces an effect resolving on enzyme resynthesis. Transcriptional downregulation, the bupropion case, produces an effect whose onset and offset follow mRNA and protein turnover, taking days in each direction and persisting after the precipitant is cleared. None is categorically preferable, but the choice determines how quickly a stable exposure is reached, how a missed dose behaves, and how the system responds to a third agent.
- **Isoform selectivity.** Selectivity for CYP2D6 over CYP3A is desirable both to limit interactions and to preserve the secondary N-demethylation route. Here, the evidence is encouraging for bupropion: its downregulation is CYP2D6-specific, leaving CYP3A4 and CYP1A2 mRNA unaffected [32]. Whether CYP2B6 is similarly spared has not been tested, and Section 10.4 explains why that gap matters.
- **Absence of competing substrate behavior at the target isoform.** An agent that is itself a high-affinity CYP2D6 substrate introduces bidirectional competition. Bupropion is cleared by CYP2B6 rather than by CYP2D6, which avoids this at CYP2D6 while creating a different overlap.
- **Pharmacodynamic transparency.** The cleanest mechanistic interpretation follows from an agent without independent activity at the targets of interest. Where an agent does carry such activity, the composite profile must be specified and the attribution problem acknowledged rather than elided.

### 8.3. Alternatives to Enzyme Inhibition

Enzyme inhibition is one solution to a problem that can also be addressed at the level of the molecule. Deuteration at the site of O-demethylation exploits the primary kinetic isotope effect to slow carbon–hydrogen bond cleavage, reducing metabolic clearance; the deuterated analogue advanced into clinical development has been reported to prolong the apparent half-life of the parent compound and to permit a lower dose of the accompanying enzyme inhibitor [35]. This approach reduces, though in its current form does not eliminate, the interaction burden and the interpretive ambiguity of a pharmacodynamically active partner, and it does not by itself normalize the influence of the CYP2D6 genotype; its effect size is bounded by the isotope effect achievable at the relevant position. Prodrug approaches, alternative routes of administration bypassing first-pass extraction, and structural modification of the morphinan scaffold to reduce CYP2D6 affinity while preserving target binding constitute further strategies. The last of these has partial precedent: dimemorfan, a 3-methyl rather than 3-methoxy morphinan, is not O-demethylated to dextrorphan, retains sigma-1 affinity comparable to that of dextromethorphan, and lacks the phencyclidine-site activity associated with the metabolite [5].

## 9. CYP2D6 Allelic Variation as a Determinant of Target Engagement

### 9.1. The Structure of the Locus

The CYP2D6 gene at 22q13.2 is among the most polymorphic pharmacogenes characterized, with structural variation including whole-gene deletion, gene duplication and multiplication, and hybrid genes arising from recombination with the adjacent CYP2D7 pseudogene [41]. Functional consequences span complete loss of function (*3, *4, *5, *6), decreased function (*10, *17, *41), *normal function* (*1, *2), and increased activity through gene copy number [41]. The molecular basis of decreased function is not uniform: recombinant CYP2D6*17, common in African populations, shows generally higher Km values than CYP2D6*1 with substrate-dependent reductions in intrinsic clearance, attributed to the altered positioning of active-site residues rather than to loss of expression [42]. Because the magnitude of the deficit differs by substrate, phenotyping results obtained with different probe drugs correlate poorly in populations where this allele is frequent [42].

Activity score systems map diplotypes onto four phenotype groups, from poor through intermediate and normal to ultrarapid metabolizers [41,43]. The mapping is imperfect: activity scores are ordinal approximations of a continuous variable, phenoconversion by concomitant inhibitors is common, and the correspondence between predicted and measured activity is far from exact; a consensus harmonization process was required to standardize the terminology and to assign a genotype carrying one nonfunctional allele to the intermediate rather than normal metabolizer group [43]. For the present purpose, the measured metabolic ratio is the more informative variable, for the reasons set out in Section 7.4.

### 9.2. Population Distribution

Allele frequencies vary substantially across populations. In a synthesis of allele frequency data from more than 60,000 subjects across 173 reports, genotype-predicted poor metabolizer status ranged from 0.4 to 5.4 percent across major world populations, genotypic intermediate metabolizers from 0.4 to 11 percent, normal metabolizers from 67 to 90 percent, and ultrarapid metabolizers from 1 to 21 percent [41]. The decreased-function allele *10 reaches the high frequency in East Asian populations, shifting the distribution toward intermediate activity, while *17 and *29 are prevalent in African populations [41,42]; the composition at this locus in South Asian populations is distinct again from European, African, and East Asian patterns [44].

This variation is directly consequential for the argument of this review. If the composite pharmacological profile of dextromethorphan is determined by CYP2D6 activity, then the population in which a given exposure regimen was characterized constrains the generality of the pharmacology observed. Extrapolation of exposure–response relationships across populations with markedly different allele frequency distributions is not a minor adjustment but a change in the composition of the active moiety.

### 9.3. Consequences for Boosting Strategies

The effect of a CYP2D6 inhibitor is not uniform across the phenotype spectrum, and this follows directly from the enzymology rather than from empirical observation. In poor metabolizers, the target enzyme is absent or minimally active, so an inhibitor produces little further change; the exposure profile approximates that of the boosted condition at baseline [7,9]. In normal metabolizers, the inhibitor produces the large increases described in Section 8 [9]. In ultrarapid metabolizers, increased enzyme copy number raises the inhibitor concentration required for comparable fractional inhibition, and a fixed inhibitor dose may produce incomplete conversion [41].

A fixed-dose combination therefore delivers different composite exposures and, by extension, different target engagement profiles across the phenotype spectrum. Framing this in molecular terms rather than as a dosing problem clarifies what would be needed to address it: either titration of the inhibitor against the measured metabolic ratio or approaches that reduce dependence on the enzyme altogether, as discussed in Section 8.3.

### 9.4. Implications for Study Design

Three recommendations follow for preclinical and translational work:

- The measured metabolic ratio, not genotype alone, should be reported as the exposure descriptor in studies relating dextromethorphan exposure to molecular outcomes.
- Parent and metabolite concentrations should be quantified separately and, where possible, unbound concentrations should be quantified at the relevant compartment, rather than reporting a single analyte or dose.
- In preclinical models, comparability to the human condition should be established on metabolic rather than dose grounds, given the substantial species differences in the enzymes mediating O-demethylation.

## 10. Molecular Rationale for Combining Dextromethorphan–Bupropion with Esketamine

A question raised by the framework is whether a CYP2D6-boosted dextromethorphan combination and esketamine, both acting at the NMDA channel, are pharmacologically redundant or potentially complementary. We distinguish three evidence levels throughout this section: established evidence for the individual agents and their metabolites; model-derived comparisons from published parameters; and hypotheses about the untested three-agent combination. Only the first level is experimentally established. The proposed complementarity, temporal interaction, and three-way pharmacokinetic effects remain hypotheses requiring direct testing. No therapeutic recommendation is made.

### 10.1. Redundancy at the Channel, Divergence Elsewhere

At the NMDA receptor channel, the two exposures share rather than complement a mechanism. Esketamine and dextrorphan are uncompetitive open-channel blockers within the noncompetitive antagonist domain [2,3]. Co-exposure could increase overall channel block, but occupancy at a shared site is not evidence of mechanistic complementarity, and the magnitude or direction of the combined effect has not been measured.

The argument, if there is one, rests on three asymmetries:

First, the available kinetic comparison does not establish that dextrorphan dissociates faster than ketamine. In the cited patch-clamp series, dextrorphan and ketamine had the same reported Koff values (0.075 per second), while dextromethorphan dissociated faster (0.179 per second) [2,13]. Differences between a boosted dextromethorphan exposure and intranasal esketamine are therefore better framed in terms of compound mixture, potency, achieved concentration, route, and temporal exposure than as a proven kinetic distinction between dextrorphan and ketamine.

Second, the non-NMDA target coverage of the two is almost disjoint. A CYP2D6-boosted dextromethorphan exposure carries sigma-1 receptor engagement and serotonin and norepinephrine transporter inhibition from the parent compound and norepinephrine and dopamine reuptake inhibition and nicotinic antagonism from the bupropion component [2,34]. None of these is a property of esketamine. Conversely, ketamine metabolites have been implicated in effects not reproduced by the parent, and ketamine and its active metabolites regulate STAT3 and type I interferon signaling in human microglia [27]. The two exposures therefore populate largely non-overlapping regions of target space outside the shared channel.

Third, and the most relevant to Section 6.3, sigma-1 activation increases GluN2A and GluN2B expression, drives NMDA receptor trafficking to the cell surface, and increases the physical association between sigma-1 and GluN2 subunits [28]. A sigma-1 component sustained continuously alongside intermittent high-amplitude channel block is a molecularly coherent, and currently untested, arrangement.

Two further points of divergence deserve explicit statement because the claim that the target space is largely non-overlapping depends on them. First, ketamine blocks HCN1 channel subunits, a molecular substrate of its hypnotic action that is distinct from NMDA channel block and is not shared by the morphinans [45]. Second, the metabolite question is more consequential for esketamine than for dextromethorphan because at least one metabolite has been proposed to act independently of the channel that defines the parent compound: (2R,6R)-hydroxynorketamine has been reported to exert antidepressant-like actions in rodents without NMDA receptor inhibition, through a mechanism involving AMPA receptor activation [46,47]. The relative contribution of this pathway remains contested, and the stereochemistry matters, since the enantiomer implicated derives preferentially from (R)-ketamine; its relevance to an S-enantiomer product should not be assumed. Taken together with the sigma-1, serotonergic, noradrenergic, dopaminergic and nicotinic arms that are properties of the oral combination alone, the molecular footprints of the two exposures overlap at one channel and diverge almost everywhere else.

### 10.2. Quantifying the Two Exposures at the Shared Channel

The comparison can be taken beyond a qualitative statement of shared mechanism. (S)-ketamine binds the phencyclidine site of the NMDA receptor channel with a Ki of 0.30 microM, approximately five times more tightly than (R)-ketamine at 1.4 microM [48]. Intranasal esketamine at the approved 84 mg dose produces mean peak plasma concentrations of approximately 95 to 164 ng/mL, equivalent to 400 to 690 nM; at 56 mg, the corresponding range is approximately 302 to 492 nM [49]. Plasma protein binding is low, with 43 to 47 percent bound, so unbound peak concentrations are approximately 212 to 393 nM at 84 mg and 160 to 281 nM at 56 mg [49].

Applying the same one-site heuristic used in Section 5.1, with binding constants on both sides to keep the input type consistent, gives the model-derived comparison in Table 5.

Three qualifications apply. Table 5 uses binding constants and therefore differs from the functional inputs in Table 3, although both base-case calculations give the same ordering. The esketamine values reflect a transient peak after intranasal dosing, whereas the oral values reflect a continuous-exposure condition. The unbound-plasma proxy is uncertain for every species. The supplementary workbook exposes all inputs and allows the concentration and potency assumptions to be varied; none of these values should be interpreted as direct human brain occupancy.

Within the base-case heuristic, intranasal esketamine produces a higher peak engagement index than the continuous oral exposure, and CYP2D6 inhibition lowers the oral NMDA index. This conditional result weakens a simple redundancy argument but does not establish complementary clinical action. The remaining question is experimental: whether non-overlapping targets and temporal exposure patterns produce benefit, additive adverse effects, or no meaningful interaction.

### 10.3. A Temporal Rather than Additive Hypothesis

These asymmetries suggest that any rationale for the combination would be temporal. Intermittent esketamine produces brief glutamatergic modulation associated, in ketamine models, with eEF2 kinase inhibition, eEF2 dephosphorylation, BDNF translation, mTORC1 activation, and AMPA receptor insertion [24,25,26,50,51]. Continuous exposure to a boosted dextromethorphan combination provides a tonic profile with sigma-1 and monoaminergic components that may affect mitochondrial bioenergetics, calcium handling, receptor trafficking, and monoaminergic signaling [14,23,28].

The hypothesis worth stating is therefore one of induction and consolidation: that intermittent high-amplitude blockade initiates synaptic remodeling, while a continuous exposure carrying distinct targets supports the cellular processes required to maintain it. We emphasize that this is a hypothesis constructed from the molecular literature, not an inference from data on the combination, and that a simpler alternative, straightforward additivity at a shared channel with additive tolerability burden and no mechanistic gain is fully consistent with what is currently known.

### 10.4. The Enzymatic Intersection: CYP2B6 and CYP3A4

One direction has been measured: repeated intranasal esketamine did not alter bupropion or hydroxybupropion pharmacokinetics in a dedicated interaction study [49], arguing against the appreciable induction or inhibition of CYP2B6 by esketamine at the studied dose. The reverse direction and the three-agent case have not been measured.

Intranasal administration bypasses hepatic first-pass extraction, and published CYP2B6 genotype findings for ketamine are inconsistent between in vitro and single-dose in vivo studies [52,53]. The appropriate conclusion is therefore that the overlap defines a testable pharmacokinetic question, not an established three-way interaction.

Dextromethorphan N-demethylation, bupropion hydroxylation, and esketamine N-demethylation involve CYP2B6 to different degrees, while CYP3A also contributes to dextromethorphan and esketamine clearance [8,19,52,53,54,55]. This is pathway overlap, not evidence that clinically meaningful substrate competition occurs. Quantitative interaction claims require relevant inhibitor or competition constants, hepatic exposure estimates, and direct pharmacokinetic observations. Section 11 provides the detailed evidence and limitations.

### 10.5. Constraints and Safety Considerations

Several considerations constrain the hypothesis and should be stated alongside it rather than in a closing caveat.

Because the compounds share an NMDA channel mechanism, co-exposure could increase channel-related adverse effects, such as dissociation, sedation, dizziness, and transient blood pressure elevation, although combined effects have not been quantified; these events are frequently reported in esketamine trials [56,57]. The bupropion component also lowers seizure threshold. Any pharmacokinetic interaction affecting exposure could alter these liabilities as well as intended effects. A fixed-dose boosted combination already produces different composite exposures across CYP2D6 phenotypes, and adding an agent that uses overlapping metabolic pathways may add further interindividual variability [31,41].

For these reasons, the combination should be treated as a question for controlled investigation with dense pharmacokinetic sampling and genotyping at both loci and not as an extrapolation to be made from the separate evidence for each agent.

### 10.6. Testable Predictions

The framework yields predictions that can be evaluated without first administering the combination clinically:

- A direct enzyme expression experiment would treat human hepatocytes with bupropion and its circulating metabolites at the concentrations used in [32] and measure CYP2B6 mRNA and protein alongside CYP2D6, CYP3A4, and CYP1A2. This would test whether the potential esketamine interaction is limited to substrate competition or could include a multi-day change in enzyme expression.
- Reversible inhibition and substrate competition parameters should be measured directly for dextromethorphan, bupropion, esketamine, and their relevant metabolites at CYP2B6 in human liver microsomes and recombinant enzymes. The resulting parameters could support a physiologically based model before clinical co-exposure is studied.
- **Genotype dependence is predictable and testable.** The magnitude of interaction should depend on CYP2B6 genotype and, for the dextromethorphan component, on CYP2D6 genotype, with the largest deviations in individuals carrying reduced-function alleles at both loci.
- **The induction-and-consolidation hypothesis is separable.** If the temporal argument of Section 10.2 holds, sigma-1 antagonism should attenuate the contribution of the continuous component without affecting the acute effects of esketamine, which is testable in preclinical models. If instead the relationship is simple additivity at the channel, the combination will show no molecular signature beyond the sum of the two exposures and no dissociation between components in eEF2, BDNF, mTORC1 and AMPA surface expression measures.

**Figure 5:**
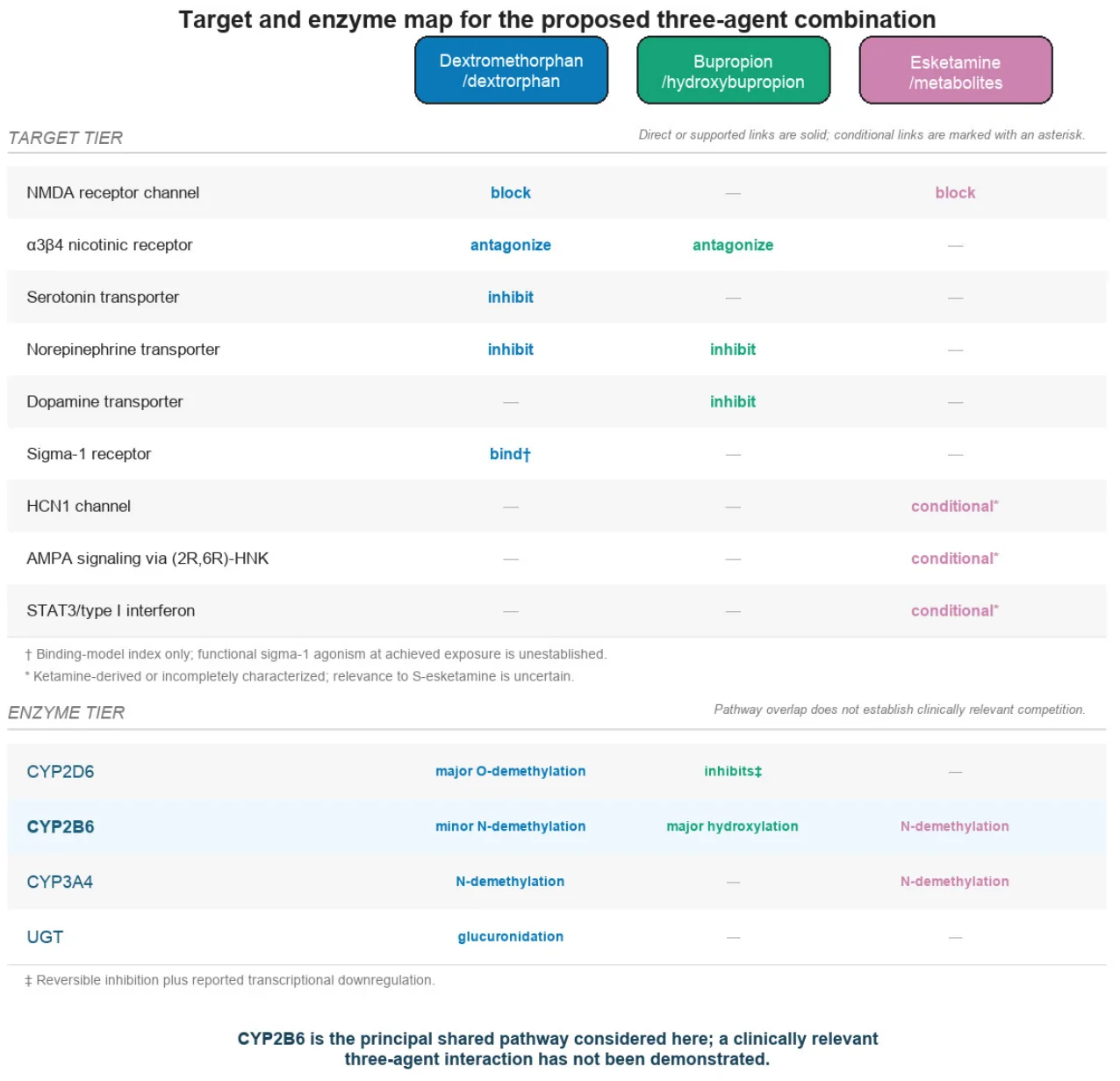
Target and enzyme map for the three administered agents and their metabolites. Dextromethorphan/dextrorphan and esketamine share NMDA-channel block; dextromethorphan and bupropion share alpha3beta4 nicotinic-receptor and norepinephrine-transporter effects. Bupropion is not depicted as an NMDA-channel blocker. HCN1, hydroxynorketamine/AMPA, and STAT3/type I-interferon links are conditional or ketamine-derived, and their relevance to S-esketamine is uncertain. CYP2B6 is a shared pathway, but pathway overlap does not establish clinically relevant substrate competition. HNK, hydroxynorketamine; UGT, UDP-glucuronosyltransferase.

Figure 5 summarizes the molecular targets and metabolic pathways shared by the three administered agents. The comparative molecular map is summarized in Table 6.

## 11. Enzymatic Interactions and Pharmacokinetic Constraints

The three administered agents involve overlapping hepatic pathways, but shared metabolism does not by itself establish substrate competition, enzyme saturation, or altered clinical exposure. The evidence below separates documented pathway assignment from model assumptions and untested interaction hypotheses. No clinically important three-way CYP2B6 interaction has been demonstrated.

### 11.1. CYP2B6 Pathway Overlap

Bupropion is the prototypical substrate for CYP2B6, and its principal active metabolite, hydroxybupropion, is formed almost exclusively by this isoform: in microsomes containing expressed enzymes, the reaction was mediated only by CYP2B6, a CYP2B6 inhibitory antibody produced more than 95 percent inhibition in human livers, and CYP2B6 content correlated with hydroxylation activity at r = 0.96 [38]. Reported Km values for the reaction lie between approximately 35 and 89 microM and the Vmax corresponds to substantial hepatic extraction [55].

Dextromethorphan undergoes N-demethylation via CYP3A4 as a lower-affinity secondary pathway with Km in the range of 230–650 microM. Substantial contributions from CYP2B6 have been documented, with reported Km values of approximately 450 microM for the CYP2B6-catalyzed reaction. While lower-affinity than CYP2D6-mediated O-demethylation (Km ~7 microM), CYP2B6 becomes proportionally more important when CYP2D6 is inhibited by bupropion, shifting the metabolic burden from high-affinity O-demethylation to lower-affinity N-demethylation.

Esketamine clearance involves both CYP2B6 and CYP3A4, with the relative contribution disputed in the literature. Microsomal work has suggested CYP3A4 predominance, whereas kinetic analysis with genotyped human liver microsomes and selective inhibitors identified CYP2B6 as the high-affinity pathway (Km approximately 22 microM) and assigned it the major role at clinically relevant concentrations. In vivo, a single low oral dose of ketamine in CYP2B6*6 carriers (reduced-function allele) showed no measurable effect on clearance, a discrepancy between in vitro prediction and clinical observation that highlights the limitations of extrapolation.

### 11.2. Substrate Competition: Mechanism and Magnitude [8,19,54,55]

When dextromethorphan, bupropion, and esketamine are co-administered, their pathways converge at CYP2B6 to different degrees. This makes interaction mechanistically possible but does not show that the compounds compete at clinically relevant hepatic concentrations. The fraction of CYP2B6 activity used for bupropion hydroxylation cannot be inferred from pathway assignment or intrinsic clearance alone [8,19,54,55].

Potential CYP2B6 substrate competition cannot be inferred from pathway overlap alone. For illustration, the reported unbound bupropion concentration (~0.3 microM) is substantially below its reported CYP2B6 hydroxylation Km (~35 microM); the simple ratio C/(Km + C) is therefore approximately 0.9%, which does not support an assumption that CYP2B6 is saturated by bupropion. However, plasma concentrations do not necessarily represent unbound concentrations at the hepatic enzyme site, and Km values alone do not establish competitive inhibition among substrates. Dextromethorphan N-demethylation (reported Km ~450 microM) and esketamine N-demethylation (reported Km ~22 microM in one experimental system) may share CYP2B6, but the magnitude and clinical relevance of any competition remain unknown. Direct measurement of interaction parameters at relevant concentrations would be needed before making quantitative predictions.

### 11.3. Expression-Level Interactions: Bupropion-Mediated CYP2B6 Downregulation?

Bupropion and its metabolites selectively downregulate CYP2D6 mRNA in human hepatocytes and primary hepatocytes in a concentration-dependent manner, with EC50 values of 0.21 microM for bupropion and 0.20 microM for S,S-hydroxybupropion, achieving maximal reductions of 78 to 88 percent. The mechanism is transcriptional rather than translational or post-translational, and it does not proceed through the small heterodimer partner (SHP) pathway by which retinoic acid represses CYP2D6. Critically, CYP3A4 and CYP1A2 mRNA were unaffected in these experiments, indicating selectivity for CYP2D6 [32]. The 2021 corrigendum to Reference [32] corrected expression-level data; numerical estimates from the original figures should therefore not be interpreted independently of that correction.

One direction of this question has already been answered, and the answer changes what remains open. In a dedicated phase 1 drug interaction study conducted during the esketamine development program, thirteen healthy subjects received oral bupropion at 150 mg on days 1 and 19 with intranasal esketamine at 84 mg on days 4, 7, 11, 14 and 18; the plasma and urinary pharmacokinetics of bupropion and hydroxybupropion were unaffected, and hepatic CYP2B6 activity was judged unaltered [49]. Esketamine therefore neither induces nor appreciably inhibits CYP2B6 at the approved dose, and the direction esketamine to bupropion can be set aside.

The opposite direction has not been tested, and it is the one the molecular argument predicts to matter. Whether bupropion-associated downregulation extends from CYP2D6 to CYP2B6 has not been examined. This is a concrete gap: if bupropion-mediated downregulation extends to CYP2B6, the clearance of dextromethorphan via N-demethylation and esketamine via N-demethylation could be substantially reduced on a time course of days (following mRNA and protein turnover) rather than hours (following inhibitor kinetics). The onset of enzyme suppression in the cited hepatocyte experiments rose from 22–63 percent at one hour to 60–93 percent at 72 h at constant inhibitor concentrations, demonstrating a temporal signature characteristic of expression-level change.

Note that the negative clinical result above does not answer this. That study used bupropion as the probe and esketamine as the precipitant; the question here is the reverse, with bupropion as the precipitant acting on the enzyme that clears esketamine, and on a time course of days rather than hours. If CYP2B6 downregulation does occur, several consequences follow: (1) the interaction would accumulate over the first 3–7 days of combined treatment and persist for days after bupropion is discontinued; (2) any phenotypic effect would be strongest in individuals with CYP2B6 intermediate or poor metabolizer alleles (reduced baseline activity), since loss of further activity would shift them toward complete loss of function; and (3) the interaction would be mechanistically distinct from reversible active-site inhibition and more difficult to predict from standard drug–drug interaction studies.

### 11.4. CYP3A4 Involvement: Secondary Pathway Sharing

CYP3A4 contributes to dextromethorphan N-demethylation and esketamine clearance. The relative contributions of CYP3A4 and CYP2B6 to ketamine N-demethylation differ across experimental systems. An interaction at CYP3A4 could therefore affect exposure, but its magnitude in the proposed three-agent setting is unknown. The cited hepatocyte experiments reported no change in CYP3A4 mRNA following exposure to bupropion and its metabolites [32]; they did not establish the absence of all possible CYP3A4-mediated interactions.

## 12. Risk–Benefit Assessment of Three-Agent Combination

The assessment that follows is mechanistic rather than empirical, and it is organized that way deliberately. No controlled data on the three-agent combination exist, so nothing here can be an estimate of clinical benefit or harm. What can be done is to work forward from the molecular facts established in Section 3, Section 4, Section 5, Section 6, Section 7, Section 8, Section 9, Section 10 and Section 11: for each point at which the three agents converge, at a shared target or shared enzyme, the predicted consequence is stated together with the measurement that would confirm or exclude it. Every risk named below is derived from a named molecular mechanism and is falsifiable at the level of that mechanism; none is inferred from trial incidence data for the individual agents.

Three of these predictions are already constrained by the quantitative work above and should be read in that light. Serotonergic burden is the clearest, as Section 5.1 shows that CYP2D6 inhibition raises the model-derived serotonin transporter engagement index from approximately 18 to approximately 74 percent, which converts a minor component of the unboosted exposure into the dominant one. Additive channel block is the weakest, as Section 10.2 shows that the oral component contributes modestly at the NMDA channel and that boosting reduces rather than increases that contribution. And the enzymatic question is now partly answered, since esketamine has been shown not to alter CYP2B6 activity at the approved dose, leaving the bupropion-to-esketamine direction as the open one.

The combination of dextromethorphan–bupropion (tonic, oral, continuous) and esketamine (acute, intranasal, episodic) is mechanistically plausible but carries substantial risks that must be weighed against potential benefits. This section provides a comprehensive assessment.

### 12.1. Synergistic Benefits and Pharmacological Complementarity

#### 12.1.1. Distinct Target Space and Temporal Complementarity

The three agents collectively occupy largely non-overlapping regions of target space outside the shared NMDA channel mechanism. Parent dextromethorphan provides serotonin-transporter inhibition; bupropion adds norepinephrine- and dopamine-transporter inhibition and nicotinic antagonism. Dextromethorphan and dextrorphan have comparable sigma-1 binding affinity, but the model does not establish functional sigma-1 agonism at achieved exposures. Findings involving STAT3, type I interferon, HCN1, and hydroxynorketamine are largely ketamine-derived or preclinical and should be treated as conditional when extrapolated to intranasal S-esketamine.

From a temporal standpoint, an induction-and-consolidation hypothesis would require intermittent esketamine exposure to initiate plasticity-related changes and sustained oral treatment to support their persistence. The proposed eEF2-BDNF-mTORC1 and monoaminergic pathways are mechanistically plausible, whereas functional sigma-1 agonism at the modeled exposure is unestablished. No study has tested this sequence in the three-agent combination.

#### 12.1.2. Clinical Rationale

Treatment-resistant depression is commonly defined by nonresponse to at least two adequate antidepressant trials [58,59]. In separate controlled trials, intranasal esketamine (generally with an oral antidepressant in the studies cited here) and dextromethorphan–bupropion improved depressive symptoms in their respective populations [39,40,56,57]. These trials did not evaluate the three-agent combination, and their response or remission rates cannot be combined to predict benefit from the proposed regimen.

### 12.2. Overlapping Adverse Effects and Safety Concerns

#### 12.2.1. Shared NMDA Channel Liabilities

Both dextromethorphan (when metabolism is inhibited) and esketamine act as NMDA channel blockers. Esketamine can cause transient dissociation, sedation, and hemodynamic effects; the incidence and magnitude of any additive effects in the proposed combination are unknown and would depend on exposure and dose timing [13,60,61].

Esketamine commonly produces transient dissociation or perceptual changes that generally resolve during post-dose observation. The cited dextromethorphan–bupropion trials did not establish a comparable incidence of dissociation. Whether coincident peak exposure increases dissociation, sedation, cognitive impairment, or driving risk has not been studied [60,61].

Both products can increase blood pressure: esketamine produces transient post-dose increases, and the dextromethorphan–bupropion label warns that bupropion can cause hypertension. The combined pressor effect has not been quantified; a future study would need protocol-defined pre-dose and post-dose blood-pressure assessment [60,61].

#### 12.2.2. Bupropion-Specific Seizure Risk

Bupropion lowers seizure threshold in a dose-dependent manner, and seizure risk varies with formulation, dose, patient characteristics and co-medications. Estimates derived from different bupropion products should not be combined into a single incidence estimate for dextromethorphan–bupropion. NMDA receptor antagonism can have context-dependent effects on neuronal excitability, but there is no direct clinical estimate of seizure risk for the three-agent combination. A higher risk than with either component alone is possible, not established.

Patients with pre-existing seizure disorder represent an absolute contraindication. Patients with histories of seizures after head injury, significant metabolic disturbance, or exposure to pro-convulsant drugs (particularly other dopaminergic stimulants) warrant special caution [62,63,64].

#### 12.2.3. Monoaminergic Hyperactivity and Serotonin Syndrome Risk [65,66]

Serotonin syndrome is a potentially life-threatening adverse effect arising from excessive central serotonergic activity, classically associated with combinations of serotonergic drugs (SSRIs, SNRIs, tricyclic antidepressants, MAOIs, tramadol, linezolid). The hallmark triad is autonomic instability (hyperthermia, hypertension, tachycardia, diaphoresis), neuromuscular abnormalities (hyperreflexia, myoclonus, rigidity), and altered mental status (agitation, confusion, tremor) [65,66].

Bupropion primarily affects norepinephrine and dopamine pathways, whereas dextromethorphan inhibits serotonin uptake. Co-prescription with serotonergic medicines can increase the potential for serotonin toxicity, especially with potent serotonergic combinations and monoamine oxidase inhibitors. The clinical significance varies by agent and regimen; concurrent use should be assessed individually rather than treated as uniformly contraindicated. The estimates in Table 3 are model-based and are not direct human SERT occupancy measurements.

Any decision to continue, adjust or discontinue a concomitant antidepressant should be based on its specific interaction profile, prescribing information and clinical circumstances. A universal 5–7-day washout for all SSRIs is not supported; monoamine oxidase inhibitors require separate, explicit contraindication and washout consideration.

#### 12.2.4. Dissociative and Cognitive Effects over Extended Treatment

Esketamine is administered episodically under medical supervision, with at least 2 h of post-dose monitoring. Dissociative and acute cognitive effects usually resolve within the monitored period, but hazardous activities and driving should be avoided until the next day after a restful sleep.

Dextromethorphan–bupropion is self-administered chronically. The cited controlled trials do not establish the long-term cognitive safety of the three-agent combination. Long-term open-label esketamine studies have not shown progressive cognitive decline at the group level, but those data do not resolve combination-specific effects, vulnerable subgroups, or functional outcomes such as driving [39,40,67,68].

### 12.3. Pharmacokinetic and Pharmacogenomic Constraints

#### 12.3.1. CYP2D6 Phenotype-Dependent Variability [31,41]

The effect of bupropion-mediated CYP2D6 inhibition is not uniform across the CYP2D6 phenotype spectrum: [31,41]

- **Poor metabolizers (nonfunctional alleles): Baseline dextromethorphan exposure is higher and conversion to dextrorphan is lower than in normal metabolizers. Bupropion may produce less additional phenoconversion, but the magnitude for the proposed combination has not been established.**
- **Normal metabolizers (normal-function alleles): CYP2D6 inhibition is expected to increase the dextromethorphan-to-dextrorphan exposure ratio. The 18- to 30-fold estimate in Section 5 derives from quinidine datasets and should not be transferred quantitatively to bupropion.**
- **Ultrarapid metabolizers (gene duplication or multiplication): Higher baseline CYP2D6 activity may reduce parent exposure despite inhibition, but the degree of phenoconversion by a fixed bupropion dose is not established for this regimen.**

CYP2D6 phenotype can therefore alter composite exposure. Metabolic ratios or genotype could be prespecified research stratification variables, but no validated genotype- or concentration-guided dosing algorithm exists for the three-agent combination.

#### 12.3.2. CYP2B6 Polymorphism and Esketamine Clearance

The CYP2B6*6 allele (a combination of nucleotide changes) is associated with reduced in vitro enzymatic activity, producing approximately 1.5- to 2-fold reductions in esketamine/ketamine N-demethylation kinetics. In vivo relevance is debated: a single low-dose oral ketamine study in healthy volunteers with genotyped CYP2B6 showed no measurable effect of the *6 allele on ketamine or metabolite clearance, but a single dose is not adequate to detect effects on steady-state accumulation or a background of other metabolic stress (hepatic impairment, concurrent CYP2B6 substrate load).

If CYP2B6 contributes materially to both dextromethorphan and esketamine N-demethylation, reduced-function alleles could alter one or both exposures. This is a testable hypothesis; compounded elevation of both substrates has not been demonstrated clinically, and the cited single-dose study did not show a measurable CYP2B6*6 effect on ketamine clearance.

#### 12.3.3. CYP3A4 and Multi-Substrate Sharing

CYP3A4 contributes to dextromethorphan N-demethylation and esketamine clearance. Inhibitors or inducers of CYP3A4 could alter exposure, but pathway sharing alone does not establish clinically important substrate competition or its direction in the proposed combination.

### 12.4. Addiction Potential and Abuse Liability

#### 12.4.1. Dextromethorphan Abuse History

Dextromethorphan has a decades-long history of abuse, particularly in adolescents and young adults who exploit its over-the-counter availability. Abuse typically involves doses of 240 to 1500 mg (8 to 50 times the 30 mg pseudobulbar affect dose), producing intense dissociation, euphoria, and visual/auditory hallucinations (“robotripping”). Neuroadaptation occurs with repeated abuse, driving dose escalation [69,70].

The abuse potential of the dextromethorphan–bupropion product was not systematically assessed in human abuse-liability studies. Dextromethorphan abuse has been reported, and the effect of CYP2D6 inhibition on misuse risk at approved doses is not established. Individual substance-use history and access to unsupervised oral medication are therefore relevant research-safety considerations.

#### 12.4.2. Esketamine Regulatory Context

Esketamine is administered under a restricted distribution program with in-clinic dosing, post-dose monitoring, and no take-home product because of risks including sedation, dissociation, respiratory depression, abuse, and misuse [71]. These controls do not eliminate risk and do not apply to the separately prescribed oral dextromethorphan–bupropion product.

Concomitant oral dextromethorphan–bupropion does not alter the requirement for supervised esketamine administration. Any study would also need safeguards against non-medical escalation of dextromethorphan-containing products.

#### 12.4.3. Polypharmacy Risk Layering

Opioids, benzodiazepines, and other central nervous system depressants may increase sedation or respiratory-depression risk with esketamine. They are not uniformly contraindicated by the esketamine label, but concomitant use requires individualized assessment and close monitoring; no combination-specific risk estimate is available.

### 12.5. Special Populations and Contraindications

#### 12.5.1. Pregnancy and Lactation [72]

Dextromethorphan: Human pregnancy data relevant to the proposed three-agent regimen are insufficient. The former FDA pregnancy-letter categories are obsolete and should not be used to characterize the risk of the combination. The compound has negligible clinically relevant mu-opioid agonism.

Bupropion: Observational pregnancy findings are heterogeneous and do not establish a specific congenital malformation risk for the proposed combination. Decisions about exposure require assessment of the available human data and the risks of untreated maternal illness.

Esketamine: Adequate pregnancy safety data for the proposed regimen are lacking. Animal developmental-toxicity findings and the known pharmacology warrant avoidance of the experimental combination during pregnancy; the relevant current product labeling should be consulted.

Lactation: Evidence on infant exposure and clinical outcomes differs substantially among the individual agents. It is not established that all three are present in breast milk at concentrations producing CNS effects in infants. The safety of the proposed combination during breastfeeding has not been studied.

Clinical implication: The proposed three-agent combination should not be initiated during pregnancy or breastfeeding outside an appropriately approved research protocol; this is a precautionary judgment about an untested regimen, not a newly established product-specific contraindication.

#### 12.5.2. Cardiac Patients

Esketamine can cause transient blood-pressure increases that peak after dosing and may persist for several hours; bupropion can also cause hypertension. The three-agent pressor effect has not been quantified. Current esketamine labeling uses a pre-dose blood pressure above 140/90 mmHg as a general guide for individualized risk assessment rather than an absolute threshold [60,61].

Esketamine is contraindicated when an increase in blood pressure or intracranial pressure poses a serious risk, including aneurysmal vascular disease, arteriovenous malformation, or a history of intracerebral hemorrhage. Other cardiovascular exclusions, ECG testing, or specialist review should be justified prospectively by the study protocol rather than presented as established combination-specific requirements [60,61].

#### 12.5.3. Seizure Disorders [62,63,64]

Bupropion is absolutely contraindicated in patients with known seizure disorders. NMDA antagonists produce context-dependent effects on seizure threshold; while they suppress excitotoxic seizures, they can paradoxically enhance susceptibility in certain network contexts (particularly in temporal lobe epilepsy models). The combined seizure risk of bupropion–dextromethorphan–esketamine is not quantified but likely exceeds that of bupropion alone [62,63,64].

#### 12.5.4. Renal and Hepatic Impairment

Dextromethorphan–bupropion: Current labeling recommends one tablet once daily in moderate renal impairment (eGFR 30–59 mL/min/1.73 m^2^) and does not recommend use in severe renal impairment (eGFR 15–29). No dose adjustment is recommended for mild or moderate hepatic impairment; use is not recommended in severe hepatic impairment. These product-specific instructions should not be replaced by doses extrapolated from other bupropion formulations.

Esketamine: In moderate hepatic impairment, exposure and terminal half-life are increased and longer post-dose monitoring may be needed. Use in severe hepatic impairment is not recommended. The proposed combination has not been evaluated in hepatic impairment.

Renal impairment: Product-specific labeling should guide eligibility and dosing. Moderate renal impairment changes the recommended dextromethorphan–bupropion schedule, whereas combination-specific pharmacokinetic and safety data are absent.

### 12.6. Drug–Drug Interactions Beyond Cytochrome P450

#### 12.6.1. Monoamine Oxidase Inhibitors

Absolute contraindication. Bupropion’s dopamine and norepinephrine reuptake inhibition could precipitate hypertensive crisis, seizures, and serotonin syndrome in the presence of MAOI activity. A minimum 14-day washout from MAOIs is required before starting bupropion–dextromethorphan–esketamine. Conversely, if an MAOI is required, dextromethorphan–bupropion–esketamine must be discontinued at least 14 days before MAOI initiation [65,66].

#### 12.6.2. Serotonergic Agents

Interaction requiring individualized assessment and monitoring. Concomitant SSRIs, SNRIs, tricyclic antidepressants, tramadol, linezolid, and other serotonergic agents can increase serotonin-toxicity risk with dextromethorphan. They are not uniformly contraindicated; monoamine oxidase inhibitors are addressed separately [65,66].

The selection of any concomitant antidepressant or anxiolytic requires individualized interaction assessment. Buspirone has serotonergic 5-HT1A activity and should not be described as non-serotonergic; mirtazapine also has clinically relevant serotonergic receptor actions despite lacking conventional SERT inhibition. Neither is an automatically safe augmentation strategy for this untested combination.

#### 12.6.3. Tramadol and Tapentadol

Strong caution: the mechanisms differ. Tramadol combines mu-opioid receptor agonism with serotonin and norepinephrine reuptake inhibition, creating serotonergic and seizure threshold concerns when combined with dextromethorphan–bupropion [73]. Tapentadol combines mu-opioid receptor agonism with norepinephrine reuptake inhibition; dopamine reuptake inhibition is not an established component of its mechanism [74]. With either agent, additive opioid and central nervous system effects require individual assessment. If opioid analgesia is necessary, an agent without monoamine reuptake inhibition may reduce pharmacodynamic overlap, with monitoring appropriate to the chosen opioid.

#### 12.6.4. Lithium

Lithium has a narrow therapeutic window, but no direct human evidence defines a specific interaction with the three-agent combination. If lithium is permitted in a study, serum concentrations and renal function should be monitored according to the protocol, the lithium product information, and clinical circumstances; a combination-specific schedule has not been validated.

#### 12.6.5. QT-Prolonging Agents

The dextromethorphan–bupropion label reports no clinically relevant QT prolongation at the maximum recommended dose, and esketamine is not labeled as a direct QT-prolonging drug. Concomitant QT-prolonging medicines, electrolyte abnormalities, and cardiac disease should be handled through individualized risk assessment; no combination-specific ECG schedule has been validated.

### 12.7. Tolerability, Discontinuation, and Withdrawal

#### 12.7.1. Tolerance Development [68,75]

Dextromethorphan and esketamine: Tolerance to dissociative effects is well documented in chronic abuse contexts, developing over weeks to months with escalating use. In clinical settings at therapeutic doses, tolerance to analgesia (a ketamine effect not relevant here) is common, but tolerance to dissociative effects and antidepressant-relevant signaling is less well characterized. Phase 2/3 trials of dextromethorphan–bupropion examined efficacy durability only to 12 weeks, insufficient to determine whether tolerance develops.

The combination of continuous dextromethorphan–bupropion and episodic esketamine introduces the possibility of differential tolerance: esketamine’s intermittent high-amplitude NMDA blockade may maintain antidepressant efficacy through continuous neuroplasticity signaling, while tachyphylaxis to dissociative effects could occur at both agents’ dissociative sites. Empirical data are needed.

#### 12.7.2. Discontinuation Syndrome

Bupropion: Abrupt cessation may be followed by recurrence or worsening of the treated condition. The cited evidence does not establish a combination-specific withdrawal syndrome or a mandatory taper duration.

Esketamine: Acute post-dose effects generally resolve within hours. Discontinuation may be followed by depressive relapse; the available evidence does not justify a categorical statement that physical withdrawal cannot occur in every patient.

Dextromethorphan–bupropion: No combination-specific discontinuation syndrome or taper schedule is established. Any discontinuation plan should follow current product information and individual clinical circumstances.

#### 12.7.3. Addiction and Psychological Dependence

Dextromethorphan: Non-medical high-dose use and psychological dependence have been reported. The determinants of misuse at approved dextromethorphan–bupropion doses are not established and should not be inferred from trauma history without supporting data.

Bupropion: Bupropion is not a scheduled substance, and no formal withdrawal syndrome is recognized. However, patients may report increased depression, fatigue, and anhedonia if bupropion is discontinued abruptly.

Combined risk: Layered misuse risk is plausible but unquantified. A future protocol should use a defined substance-use risk assessment and proportionate safeguards rather than assume that a specific behavioral-dependence mechanism has been demonstrated.

### 12.8. Monitoring Domains for a Future Controlled Study

A future controlled study of the three-agent combination would require intensive safety and exposure monitoring and safeguards against misuse. The following domains are research protocol considerations, not clinical prescribing instructions:

#### 12.8.1. Baseline Assessment [43,76]

- Comprehensive psychiatric evaluation, including assessment of suicidal ideation, homicidal ideation, psychotic symptoms;
- Neurological examination, including seizure history (personal and family), history of head injury or TBI, and baseline cognitive screening (Mini-Cog or MMSE);
- Cardiac evaluation: blood pressure, heart rate, baseline ECG (to assess QT, PR, QRS intervals), cardiac history;
- Metabolic panel (electrolytes, glucose, renal function, hepatic function, lipid panel);
- Urinalysis and urine drug screen;
- Pregnancy test if applicable;
- CYP2D6 and CYP2B6 genotyping (optional but recommended for risk stratification);
- Substance use disorder screening (OARRS/PDMP query if available; AUDIT-C for alcohol, DAST-10 for drugs);
- Driving assessment or cognition baseline (if available).

#### 12.8.2. Ongoing Monitoring During Treatment

- Psychiatric: Suicidality/homicidality assessment should be conducted at each visit, as well as monitoring of mood, anxiety, sleep quality, functional status, and adherence.
- Seizure precautions: Any reported seizure-like activity warrants immediate evaluation; patients should be educated on seizure warning signs.
- Cognitive/driving: A protocol may include baseline and periodic assessment (for example, monthly for 3 months and then every 3 months), but no validated combination-specific schedule or test battery exists.
- Vital signs: Blood pressure and heart rate should be monitored at each dextromethorphan–bupropion prescription refill, as well as full vital signs before each esketamine session.
- Substance use: Structured inquiry into any non-medical dextromethorphan use, alcohol consumption, or other drug use should be conducted; random UDS may be warranted in high-risk individuals.
- Renal/hepatic function: Renal/hepatic function should be monitored at baseline and annually, or if symptoms are suggestive of organ dysfunction development.
- Laboratory monitoring during esketamine sessions: None is standardized; vital sign monitoring is as per protocol.

#### 12.8.3. Pharmacogenomic Testing and Genotype-Based Dosing

- CYP2D6 genotyping or phenotyping may be considered as a research stratification variable; its utility for guiding this untested three-agent regimen has not been established [31,41].
- CYP2B6 genotyping is a research-level consideration; it does not provide a validated esketamine dose adjustment algorithm for the proposed regimen.
- Genotype-based dose adjustments for the three-agent combination remain to be developed and prospectively validated. The following are research considerations, not prescribing instructions:
- CYP2D6 normal metabolizers: Measure dextromethorphan and dextrorphan exposure in any approved study protocol; do not infer an individualized three-agent dose from genotype alone.
- CYP2D6 intermediate metabolizers: Quantify parent-to-metabolite exposure and tolerability prospectively rather than assuming a validated bupropion dose reduction.
- CYP2D6 poor metabolizers: Expect altered parent-to-metabolite exposure; no evidence-based dextromethorphan or bupropion dose for this untested three-agent regimen can be specified.
- CYP2B6*6 carriers: Do not infer a dose adjustment from the current evidence; collect exposure and outcome data prospectively in any approved study.

#### 12.8.4. Therapeutic Drug Monitoring (TDM)

- Dextromethorphan and dextrorphan: Assays are available. Therapeutic drug monitoring is not standard and should be considered a research exposure measure, not a validated basis for dose adjustment in this combination.
- Bupropion/hydroxybupropion: Assays are available; TDM could confirm CYP2B6 activity or detect non-adherence.
- Esketamine/norketamine: The drugs are not routinely measured; research-level TDM could provide phenotype-specific exposure data.
- Integrated TDM (all three agents + metabolites at peak and trough) is research-level and could support PBPK modeling

#### 12.8.5. Abuse Diversion Screening and Risk Mitigation

- Prescription-monitoring database review: Use should follow applicable law and protocol-defined risk safeguards; requirements vary by jurisdiction.
- Medication accountability: Pill counts or dispensing controls may be used when proportionate to protocol-defined risk.
- Urine drug testing: Frequency and analytes should be protocol-defined; the evidence does not support a universal one- to two-times-monthly schedule.
- Dispensing safeguards: Single-prescriber arrangements or limited dispensing may be considered when required by law or justified by an individual risk assessment.
- Patient education: Explicit counseling about dextromethorphan abuse risk, non-medical use, interaction with opioids/benzodiazepines is recommended.

#### 12.8.6. Cognitive and Driving Safety Assessment

- Baseline and follow-up cognitive testing may be prespecified for research purposes; no combination-specific schedule or instrument has been validated.
- Driving: The current esketamine product instructions, including avoiding driving or hazardous activities until the following day after a restful sleep, should be followed. The effects of combining esketamine with dextromethorphan–bupropion on driving performance have not been studied.
- Cognitive testing should be repeated if the patient reports subjective cognitive complaints or if a driving incident occurs.
- Driving simulation (research-level) could quantify actual impairment.

### 12.9. Summary Risk–Benefit Judgment

#### 12.9.1. Hypothesized Potential Benefits

- Mechanistic complementarity: tonic monoaminergic + sigma-1 (dextromethorphan–bupropion) + acute glutamatergic + microglial immune (esketamine);
- Temporal induction–consolidation: episodic plasticity trigger (esketamine) supported by continuous neuromodulation (dextromethorphan–bupropion);
- Hypothetical improved efficacy in treatment-resistant patients failing mono-agents;
- Potentially lower relapse risk through multi-target engagement.

#### 12.9.2. Substantial Risks

- Additive dissociative, sedative, cognitive toxicity (unknown magnitude, inadequately studied);
- Seizure risk from bupropion + potential NMDA antagonist contribution (not quantified);
- Abuse potential of dextromethorphan outside supervised clinic setting;
- Pharmacokinetic complexity: CYP2D6, CYP2B6, CYP3A phenotypes each introduce interindividual variability; fixed-dose regimen poorly suited;
- Extensive drug interaction risk (serotonergic agents, tramadol, MAOIs, lithium, QT prolongers, opioids) [65,66];
- Pregnancy or breastfeeding: investigational exposure outside an approved research protocol should be avoided; combination-specific data are lacking;
- Cardiac concerns in hypertensive or ischemic patients.

#### 12.9.3. Overall Assessment

The combination is mechanistically plausible but untested. This review does not support therapeutic use or provide prescribing instructions. Evaluation should occur only in an appropriately approved study with prospectively defined eligibility, monitoring, stopping rules, and informed consent. Controlled comparative data are required before any conclusion about benefit, harm, or clinical role.

## 13. Research Protocol Eligibility Considerations for an Untested Combination

This section does not define clinical indications or establish combination-specific contraindications. It maps potential exclusions and monitoring variables for future ethics-approved studies. Current product labels and local regulatory requirements for each individual agent remain controlling.

### 13.1. Potential Research Protocol Exclusions

- Uncontrolled seizure disorder or history of seizures not controlled on anticonvulsant medication [62,63,64];
- Current use of MAOI or use within 14 days prior [65,66];
- Renal function incompatible with current product labeling or the protocol-defined dosing plan; current dextromethorphan–bupropion labeling does not recommend use at eGFR 15–29 mL/min/1.73 m^2^;
- Decompensated cirrhosis or Child–Pugh C liver disease;
- Blood pressure or vascular disease for which an esketamine-associated increase would pose a serious risk; a pre-dose value above 140/90 mmHg is a general guide for individualized assessment, not an absolute universal exclusion [60,61];
- Unstable cardiovascular disease, defined prospectively and justified by the protocol rather than by an unsupported fixed 6-month interval;
- Pregnancy (until adequate safety data);
- Breastfeeding;
- Known hypersensitivity/allergy to dextromethorphan, bupropion, esketamine, or excipients;
- Concurrent use of other NMDA antagonists (for abuse prevention).

### 13.2. Additional Risk Stratification Considerations

- Personal or family history of bipolar disorder with prior manic/hypomanic episodes on stimulants (risk of mood switching with bupropion’s dopaminergic activity);
- Uncontrolled anxiety disorder or phobic disorder (NMDA antagonism may exacerbate dissociative symptoms) [60,61];
- Active substance use disorder or other misuse risk that cannot be managed safely under the protocol; no universal remission interval is established;
- Hepatic impairment: no dextromethorphan–bupropion dose adjustment is recommended for Child–Pugh A or B; moderate impairment may require longer esketamine monitoring;
- Moderate renal impairment (eGFR 30–60 mL/min/1.73 m^2^);
- History of psychosis or psychotic disorder (NMDA antagonism may precipitate psychotic relapse);
- Dementia or significant baseline cognitive impairment (dissociative and cognitive effects of combination would be poorly tolerated);
- Uncontrolled diabetes (bupropion may elevate blood glucose).

### 13.3. Illustrative Enrollment Variables for a Future Study

A future ethics-approved study would need prospectively defined eligibility variables, such as:

- **Diagnosis: protocol-defined treatment-resistant major depressive disorder using a validated adequacy criterion; any acute-suicidality population should be specified separately;**
- **Age: an adult age range justified by the study question and available safety data; the upper limit should be protocol-defined rather than assumed to be 75 years;**
- **Medical stability: protocol-defined exclusions for conditions that materially increase risk, without requiring absence of all comorbidity [62,63,64]**;
- **Baseline cognition: a validated measure and threshold selected prospectively for the study population; no combination-specific MMSE cutoff is established;**
- **Medication naivety to esketamine:** The study should include patients not currently on esketamine (combination specifically designed for polypharmacy synergy, not for augmentation of esketamine alone).
- **Adherence and attendance: ability to follow the protocol and attend the product-label-consistent esketamine schedule selected for the study;**
- **Capacity for informed consent:** Patients should be able to understand the risks (particularly dissociation, seizure, abuse potential) and benefits and be able to commit to intensive monitoring.
- **Substance-use history: a protocol-defined assessment of current disorder and misuse risk, without an unsupported universal 1-year remission rule;**
- **Social support:** Patients should preferably have reliable access to transportation, family/caregiver support, and the capacity to report adverse effects.
- **No planned pregnancy:** Patients should be willing to use contraception or confirm they are not pregnant.
- **Driving and transport: ability to avoid driving or hazardous activities until the day after each esketamine session following a restful sleep, with safe transportation from the clinic;**

### 13.4. Participant Information Topics for a Future Research Protocol

Before enrollment and investigational exposure, participants should receive protocol-specific information on:

- **Dissociation and cognitive effects,** including what to expect, duration, and safety precautions (avoid driving/machinery);
- **Seizure risk,** including warning signs of seizure activity (staring spells, loss of consciousness, rhythmic jerking) and an emergency action plan;
- **Dextromethorphan abuse potential,** including risk of non-medical escalation and contraindication with other NMDA antagonists or “cough medicines” containing dextromethorphan;
- **Drug interactions, including the MAOI contraindication and individualized assessment of serotonergic agents, tramadol, lithium, opioids, benzodiazepines, and other central nervous system depressants [65,66];**
- **Monitoring intensity, including protocol-defined visits, laboratory testing, substance-use safeguards, and cognitive assessments;**
- **Pregnancy prevention,** including effective contraception if the patient is of childbearing potential;
- **Alcohol and substance use,** including the absolute avoidance of alcohol during esketamine sessions and strong caution regarding other CNS depressants.

## 14. Limitations

The limitations of this review are stated together rather than dispersed because several of them bear on the same conclusions.

**Search and selection.** A single bibliographic database was searched, with filters restricting results to 2010–2026, the English language and human studies; primary pharmacology predating 2010 therefore entered the review through citation tracking rather than through the queries themselves. No formal risk-of-bias assessment or certainty-of-evidence grading was applied, and no pooled estimate was generated. Screening and selection were not conducted in duplicate by independent reviewers. The source selection diagram is not a PRISMA flow diagram and should not be read as one.

The unbound-plasma assumption. All potency–exposure comparisons and all model-derived engagement indices assume that unbound plasma concentrations approximate brain interstitial concentrations. This assumption is inherited from the compilation in [2] and is not independently validated here. It is the least secure for dextrorphan, which is more polar than the parent and extensively glucuronidated, and the direction of any error is unknown.

**Heterogeneity of the source data.** The affinities and potencies compared in Table 1, Table 3 and Table 6 derive from different species, preparations, radioligands and functional readouts. Comparison between the two exposure regimens within a single target is more secure than comparison across targets.

The one-site model. Uncompetitive open-channel block is not described fully by an equilibrium one-site equation. Use dependence, trapping, open-channel probability, subunit composition, and temporal exposure are not captured. The NMDA values in Table 3 and Table 5 are model-derived engagement indices and should be read as conditional ordering and direction-of-change estimates, not as absolute receptor occupancy. The supplementary sensitivity analysis shows that the base-case direction is stable to individual 0.5-fold to 2-fold perturbations but can reverse under an extreme joint shift in relative parent and metabolite assumptions.

**Single pharmacokinetic dataset.** The parent-to-metabolite ratios that drive the analysis of Section 5 derive from one pharmacokinetic study. The estimates inherit its uncertainty, and no propagation of that uncertainty has been performed.

**Quantification of the esketamine component.** Section 5 estimates the target engagement for the oral dextromethorphan component, while Section 10.2 and Table 5 extend the illustrative one-site calculation to intranasal esketamine using published binding constants and estimated unbound peak plasma concentrations. These calculations are not direct measurements of brain receptor occupancy. The oral and intranasal estimates also represent different sampling conditions and temporal exposure profiles; accordingly, their numerical comparison should be interpreted cautiously and should not be taken as evidence of equivalent central exposure or clinically additive effects. The cited Ebert et al. Ki of 0.30 micromolar was measured in rat-derived receptor preparations, not by direct human brain occupancy imaging; cross-species extrapolation is uncertain.

**Sigma-1 functional status.** The gap between occupancy and functional agonism is unresolved throughout. Every statement depending on sigma-1 signaling is conditional and marked as such.

**Absence of data on the combination.** No controlled data exist. The analysis in Section 10, Section 11, Section 12 and Section 13 is constructed from the properties of the individual agents and is a framework for investigation, not evidence of effect.

## 15. Research Agenda and Future Directions

Several gaps are sufficiently well defined to be stated as researchable questions:

**Target engagement of the composite exposure has not been measured.** No study has systematically characterized receptor occupancy at NMDA, sigma-1, and monoamine transporter sites as a function of a defined parent-to-metabolite ratio. Positron emission tomography approaches exist for transporters and are under development for sigma-1; combining these with the controlled manipulation of CYP2D6 activity would convert the argument of Section 5 from inference to measurement.

**The relative contribution of the three arms is unquantified.** Selective pharmacological or genetic ablation of the sigma-1 receptor, the transporter component, and NMDA channel block in the same experimental system would establish which arm carries which molecular outcome. The available literature addresses these largely in isolation.

**3-Methoxymorphinan is uncharacterized.** This metabolite becomes a proportionally larger component of the metabolite pool precisely under the conditions created by CYP2D6 inhibition, and its target profile has not been determined with modern methods. This is a tractable and well-bounded piece of work.

**Brain exposure is inferred rather than measured.** The relationship between plasma concentrations of the parent and metabolite and their unbound concentrations in brain tissue, including the influence of efflux transporters and glucuronidation, has not been established in a way that supports a quantitative inference about central target engagement.

**Sigma-1 and NMDA interaction remains a hypothesis.** The physical and functional association between the two systems, and the possibility of supra-additive signaling under composite exposure, is mechanistically plausible and experimentally untested in this context.

**The molecular basis of interindividual variability extends beyond CYP2D6.** UGT2B15 and UGT2B7 polymorphism, sigma-1 receptor variants, and glutamate receptor subunit variants all plausibly modulate response, and none has been examined systematically in relation to dextromethorphan pharmacology.

A productive framing for this program is that dextromethorphan should be treated not as a drug with a metabolite but as a two-component system whose composition is experimentally controllable. Once the system is described in those terms, the relevant experiments become straightforward to specify.

## 16. Conclusions

Dextromethorphan and dextrorphan occupy differently weighted positions within the same target space. The parent compound is more potent at serotonin reuptake inhibition and less potent at NMDA channel block, whereas dextrorphan shows the opposite weighting. In the cited binding assay, the sigma-1 affinities are comparable for the two species; sigma-1 engagement therefore depends on their combined unbound exposure rather than an intrinsic parent–metabolite affinity difference. Because interconversion is governed by a highly polymorphic enzyme, CYP2D6 activity determines both total exposure and the mixture of pharmacological species present.

This reframes pharmacokinetic boosting as something other than an exposure enhancement technique, and the two established combinations turn out to work by different mechanisms—potent reversible active-site occupancy with quinidine and the predominantly transcriptional downregulation of the enzyme with bupropion—with different time courses and different consequences when a third agent is introduced. Inhibiting CYP2D6 shifts the composition of the active moiety toward the parent compound, changing the ratio of free parent to free metabolite by a factor of approximately 90, and thereby strengthens the serotonergic component, which is weak in the unboosted exposure. The established combinations with quinidine and with bupropion are best understood as two solutions to this compositional problem, differing in whether the enzymatic function is delivered by a pharmacodynamically silent agent or by one contributing its own target profile. Stated at the level of enzyme kinetics, the requirement for such a partner is specifiable independently of therapeutic class: sufficient potency to act below its own pharmacological threshold, a defined inhibition mechanism, selectivity for CYP2D6 over the secondary pathway, and absence of competing substrate behavior.

The principal gaps are measurement gaps. Target engagement has not been quantified as a function of the parent-to-metabolite ratio, the contributions of the three pharmacological arms have not been dissociated, the minor metabolites that accumulate under enzyme inhibition remain uncharacterized, and central exposure is inferred from plasma rather than measured. Each is addressable with existing methods.

The same framework clarifies what is at stake in combining a boosted dextromethorphan preparation with esketamine. The exposures share an NMDA channel mechanism, so any complementarity would have to arise from non-overlapping targets or temporal exposure patterns rather than from distinct glutamatergic sites. CYP2B6 and CYP3A participate in the metabolic pathways of more than one component, but pathway overlap alone does not establish competition or a clinically relevant interaction. Esketamine did not alter bupropion or hydroxybupropion pharmacokinetics in the cited study [49]; the reverse direction and the three-agent case remain untested. The proposed combination should therefore be treated as a research hypothesis, not a therapeutic recommendation.

## Figures and Tables

**Table 1:** The binding affinities, functional potencies, and channel block kinetics of dextromethorphan and dextrorphan. Radioligand binding and functional potencies are reported separately because they diverge for the transporters. Unbound human exposure is tabulated separately in Table 2. Primary sources are cited by row; [2] is the compilation in which these values were assembled.

Target	Assay	Preparation	Dextromethorphan	Dextrorphan	Ref.
**Radioligand binding**					
Serotonin transporter	[3H]paroxetine, 0.2 nM	Displacement	Ki 40 nM	Ki 484 nM	[2,11]
Sigma-1 receptor	3H-pentazocine, 2 nM	Displacement	Ki 150 nM	Ki 118 nM	[2,11]
Norepinephrine transporter	[3H]nisoxetine, 2 nM	Displacement	Ki 6000 nM	Ki 6200 nM	[2]
NMDA receptor, PCP site	[3H]MK-801, 0.5 nM	Displacement	Ki 962 nM	Ki 148 nM	[2]
NMDA receptor, PCP site	[3H]MK-801, 3 nM	Displacement	Ki 2120 nM	Ki 892 nM	[2,11]
Sigma-2 receptor	[3H]DTG, 2.5 nM	Displacement	Ki 11,060 nM	Ki 11,325 nM	[2,5]
mu-, delta-, kappa-opioid	Displacement	Screening panel	No significant inhibition at 10,000 nM	Not reported	[2]
**Functional potency**					
Serotonin reuptake	[3H]5-HT uptake	Rat brain synaptosomes	Ki 23 nM	Ki 401 nM	[2,12]
Norepinephrine reuptake	[3H]NA uptake	Rat brain synaptosomes	Ki 240 nM	Ki 340 nM	[2,12]
NMDA receptor channel	Voltage clamp	Cultured mouse neocortical neurons	IC50 550 nM	IC50 72 nM	[2,17]
alpha3beta4 nicotinic	Voltage clamp	Xenopus oocytes	IC50 700 nM	IC50 1300 nM	[2,16]
alpha4beta2 nicotinic	Voltage clamp	Xenopus oocytes	IC50 3900 nM	IC50 3000 nM	[2,16]
GluN1/GluN2A NMDA receptor	Recombinant	Recombinant cells	IC50 11,000 nM	Not reported	[2]
**Channel block kinetics**					
NMDA channel, patch clamp	Voltage clamp, −70 mV	Rat superior colliculus neurons	IC50 6100 nM	IC50 1300 nM	[2,13]
NMDA channel, off-rate	Koff	Rat superior colliculus neurons	0.179 per second	0.075 per second	[2,13]
Comparators, same assay	Koff	Rat superior colliculus neurons	Ketamine: 0.075; memantine: 0.200; MK-801: 0.005 per second		[2,13]

Abbreviations: DTG, di(2-tolyl)guanidine; PCP, phencyclidine.

**Table 2:** Unbound human exposure to dextromethorphan and free dextrorphan with and without CYP2D6 inhibition. The regimens differ and are not interchangeable; the 30 mg single-dose comparison is used throughout this review for potency–exposure comparison because the parent and free metabolite were measured in the same subjects.

Regimen	Unbound Dextromethorphan	Free Unconjugated Dextrorphan	Free Parent:Metabolite	Ref.
Dextromethorphan at 30 mg alone	3.7 nM	Approximately 23.7 nM (6.4-fold above parent)	1:6.4	[2,18]
Dextromethorphan at 30 mg + quinidine	66 nM	Approximately 4.7 nM (14-fold below parent)	14:1	[2,18]
Dextromethorphan/quinidine at 20/10 mg twice daily, steady state	35–120 nM across trials	Substantially lower than parent	Not separately reported	[2,18]
Dextromethorphan at 60 mg twice daily, no inhibitor	Mean total plasma: 12 ng/mL; several subjects below limit of quantification	Predominant species	Metabolite-dominated	[9]
Dextromethorphan at 60 mg + quinidine at 75 mg, both twice daily	Mean total plasma: 241 ng/mL (approximately 20-fold increase)	Not separately reported	Parent-dominated	[9]

**Table 3:** The model-derived fractional-target-engagement index for dextromethorphan and free dextrorphan combined under 30 mg dosing with and without CYP2D6 inhibition. The values use the functional potencies from Table 1, the unbound concentrations from Table 2, and the one-site heuristic described in Section 5.1. They are calculated illustrations, not experimental measurements, in vivo receptor occupancies, or clinical effect estimates. The NMDA row is especially conditional because open-channel kinetics and use dependence are not represented. Source data: functional potencies [2,11,12,16,17] and unbound pharmacokinetic concentrations [2,18].

Target (Parent/Metabolite Potency)	Without CYP2D6 Inhibition	With CYP2D6 Inhibition	Parent Contribution to Estimated Total Engagement (Percentage Points)
Serotonin transporter (Ki 23/401 nM)	18.0%	74.2%	73.9%
Norepinephrine transporter (Ki 240/340 nM)	7.8%	22.4%	21.3%
NMDA receptor channel (IC50 550/72 nM)	25.1%	15.6%	10.1%
alpha3beta4 Nicotinic receptor (IC50 700/1300 nM)	2.3%	8.9%	8.6%
Sigma-1 receptor (Ki 150/118 nM)	18.4%	32.4%	29.7%

**Table 4:** The molecular characteristics and temporal signatures of strategies used or proposed to increase dextromethorphan exposure. The table distinguishes enzyme-directed approaches, which alter clearance, from molecule-directed approaches, which alter the substrate.

Approach	Mechanism	Potency	Isoform Selectivity	CYP2D6 Substrate?	Independent CNS Pharmacology	Effect/Time Course	Ref.
Quinidine	Reversible competitive inhibition	Ki approx. 0.1 microM against dextromethorphan O-demethylation in human liver microsomes; 0.41 microM in recombinant enzyme	High; inhibits high-affinity component accounting for approx. 96 percent of intrinsic clearance by this route.	Weak; far more potent as inhibitor than as substrate	None desired; carries cardiac potassium channel block and QT prolongation. At approved combination dose, plasma quinidine is only 1 to 3 percent of antiarrhythmic concentration.	Parent Cmax rose from 2.9 to 51 ng/mL (unbound 3.7 to 66 nM) at 30 mg dextromethorphan; half-life extended from approx. 4 to approx. 13 h; effect tracks inhibitor concentration.	[2,8,9,18,33]
Bupropion	Mixed: weak reversible inhibition plus transcriptional downregulation of CYP2D6	Reversible IC50 3 to 41 microM in human liver microsomes (S-bupropion 3, R-bupropion 41, R,R-OH 5, S,S-OH 14, threohydro 13, erythrohydro 3); downregulation EC50 0.21 microM (bupropion) and 0.20 microM (S,S-OH-bupropion), Emax 78 to 88 percent. Expression-level estimates require interpretation alongside 2021 corrigendum to [32].	Downregulation is CYP2D6-specific: CYP3A4 and CYP1A2 mRNA unaffected. Effect on CYP2B6 not tested.	No; bupropion is standard probe substrate for CYP2B6	Yes: norepinephrine and dopamine reuptake inhibition, nicotinic antagonism; lowers seizure threshold	Reversible inhibition alone predicts 43 percent enzyme inhibition; adding downregulation predicts 82 percent, against approx. 90 percent observed. Apparent inhibition in hepatocytes rose from 22 to 63 percent at 1 h to 60–93 percent at 72 h at constant inhibitor concentration.	[31,32,34]
O-demethyl deuteration	Chemical: primary kinetic isotope effect slows carbon–hydrogen bond cleavage	Not applicable	Not applicable	Yes, but turned over more slowly	None added by modification	Prolongs apparent half-life of parent; permits lower dose of accompanying inhibitor.	[35]
Morphinan scaffold modification	Removes metabolically labile 3-methoxy group	Not applicable	Not applicable	Not O-demethylated to dextrorphan	Depends on analogue; dimemorfan retains sigma-1 affinity (Ki 151 nM) without phencyclidine-site activity.	No dextrorphan formed; pharmacology is that of single species rather than mixture.	[5]
Prodrug/alternative route	Bypasses presystemic hepatic extraction	Not applicable	Not applicable	Unchanged	None added	No data identified for this scaffold.	—

**Note.** Bupropion combines weak reversible CYP2D6 inhibition with transcriptional downregulation. Whether bupropion or its metabolites similarly alter CYP2B6 expression has not been tested.

**Table 5:** The model-derived NMDA channel engagement indices for the two exposures, calculated from radioligand-binding constants. Oral values use the Table 1 binding Ki values (dextromethorphan: 962 nM; dextrorphan: 148 nM) and Table 2 unbound concentrations; esketamine values use the (S)-ketamine Ki of 300 nM [48] and unbound peak concentrations derived from the Cmax and protein binding [49]. These are calculations from published parameters, not measured brain receptor occupancy. Oral and intranasal estimates represent different sampling times and are not directly pharmacokinetically comparable. Sources: [2,11,18,48,49].

Exposure	Unbound Concentration	Model-Derived Channel Engagement Index	Temporal Pattern
Dextromethorphan at 30 mg, no CYP2D6 inhibitor	Parent: 3.7 nM; free metabolite: 23.7 nM	Approximately 14.1%	Continuous
Dextromethorphan at 30 mg + CYP2D6 inhibitor	Parent: 66 nM, free metabolite: 4.7 nM	Approximately 9.1%	Continuous
Esketamine at 56 mg intranasal, at peak	160–281 nM	Approximately 35–48%	Intermittent; tmax: 20–40 min
Esketamine at 84 mg intranasally, at peak	212–393 nM	Approximately 41–57%	Intermittent; tmax: 20–40 min

**Table 6:** A comparative molecular map of dextromethorphan, bupropion, esketamine, and their principal metabolites. The table distinguishes shared NMDA channel pharmacology from non-NMDA target divergence and identifies the involvement of CYP2B6 in the metabolic pathways of the three administered agents.

Species	Origin	Principal Target Profile	Principal Metabolizing Enzyme	Role in Combination	Exposure Pattern	Ref.
Dextromethorphan	Administered	5-HT reuptake (Ki 23 nM) > NA reuptake (240 nM) > NMDA channel (IC50 550 nM) > alpha3beta4 (700 nM); sigma-1 binding Ki 150 nM; microglial NOX2 inhibition	CYP2D6 (O-demethylation, high affinity); CYP3A and CYP2B6 (N-demethylation, low affinity)	Active moiety whose exposure combination is designed to preserve	Continuous, oral	[2,5,6,7,8]
Dextrorphan	CYP2D6 O-demethylation	NMDA channel (IC50 72 nM) > NA reuptake (340 nM) > 5-HT reuptake (401 nM) > alpha3beta4 (1300 nM); sigma-1 binding Ki 118 nM, comparable to parent	UGT glucuronidation	Principal species after unboosted oral dosing; carries glutamatergic component	Continuous, secondary to parent	[2,3,4,5]
3-Methoxymorphinan	CYP3A and CYP2B6 N-demethylation	Not characterized	Further demethylation to 3-hydroxymorphinan	None assigned; increases in proportion under CYP2D6 inhibition	Continuous, increases with boosting	[7,8,19]
3-Hydroxymorphinan	Sequential demethylation	Residual NMDA and sigma activity; neurotrophic and anti-inflammatory effects reported preclinically	Conjugation	None assigned	Continuous	[2]
Bupropion	Administered	Norepinephrine and dopamine reuptake inhibition; nicotinic antagonism; lowers seizure threshold	CYP2B6 (hydroxylation to hydroxybupropion, almost exclusively)	Enzymatic modulator (CYP2D6 inhibition) and pharmacodynamically active in its own right	Continuous, oral	[31,32,34,55]
Hydroxybupropion	CYP2B6 hydroxylation	Active; contributes to parent’s monoaminergic profile	Further conjugation	Contributes to transcriptional-downregulation-associated component of CYP2D6 inhibition	Continuous	[32,34,55]
Esketamine	Administered	NMDA receptor open-channel block at psychoactive exposures	CYP2B6 (high affinity) and CYP3A4; relative contribution contested	Active moiety; intermittent glutamatergic modulation	Intermittent, intranasal (first pass bypassed)	[52,53,54,56,57]
Norketamine and downstream metabolites	N-demethylation and hydroxylation	Metabolites implicated in effects not reproduced by parent; ketamine and active metabolites regulate STAT3 and type I interferon signaling in human microglia	CYP2B6 and CYP2A6 (hydroxylation of norketamine)	Contributes to composite effect of esketamine component	Intermittent	[27,54]

## Data Availability

No new experimental or clinical observations were collected. New illustrative calculations were performed using published source parameters. The illustrative quantitative estimates in Table 3 and Table 5 were calculated from previously published pharmacokinetic and pharmacodynamic parameters using the assumptions and equations described in Section 5.1 and Section 10.2. The corresponding calculation worksheets are provided as Supplementary Materials. These estimates are model-derived and do not represent directly measured receptor occupancy.

## Supplementary Materials

The following supporting information can be downloaded at: https://www.mdpi.com/article/10.3390/ijms272110036/s1.

## Abbreviations

The following abbreviations are used in this manuscript:

AMPA	α-amino-3-hydroxy-5-methyl-4-isoxazolepropionic acid
AUDIT-C	Alcohol Use Disorders Identification Test, consumption items
AXS-05	dextromethorphan–bupropion fixed-dose combination
BDNF	brain-derived neurotrophic factor
CNS	central nervous system
CREB	cAMP response element-binding protein
CYP	cytochrome P450
CYP1A2	cytochrome P450 1A2
CYP2B6	cytochrome P450 2B6
CYP2D6	cytochrome P450 2D6
CYP3A4	cytochrome P450 3A4
DAST-10	Drug Abuse Screening Test, 10-item version
DTG	1,3-di-o-tolylguanidine
EC50	half-maximal effective concentration
ECG	electrocardiogram
eGFR	estimated glomerular filtration rate
GluN	NMDA receptor subunit (GluN1, GluN2A, GluN2B)
HCN1	hyperpolarization-activated cyclic nucleotide-gated channel 1
IC50	half-maximal inhibitory concentration
Ki	inhibition constant
MAM	mitochondria-associated endoplasmic reticulum membrane
MAOI	monoamine oxidase inhibitor
MK-801	dizocilpine
MMSE	Mini-Mental State Examination
mTORC1	mechanistic target of rapamycin complex 1
NADPH	reduced nicotinamide adenine dinucleotide phosphate
NET	norepinephrine transporter
NMDA	N-methyl-D-aspartate
OARRS	Ohio Automated Rx Reporting System
PBPK	physiologically based pharmacokinetic
PCP	phencyclidine
PDMP	prescription drug monitoring program
PSD-95	postsynaptic density protein 95
SERT	serotonin transporter
SNRI	serotonin–norepinephrine reuptake inhibitor
SSRI	selective serotonin reuptake inhibitor
STAT3	signal transducer and activator of transcription 3
SUD	substance use disorder
TDM	therapeutic drug monitoring
TRD	treatment-resistant depression
TrkB	tropomyosin receptor kinase B
UDP	uridine diphosphate
UDS	urine drug screen
UGT	UDP-glucuronosyltransferase
